# Sensitivity and Specificity of Multiple Kato-Katz Thick Smears and a Circulating Cathodic Antigen Test for *Schistosoma mansoni* Diagnosis Pre- and Post-repeated-Praziquantel Treatment

**DOI:** 10.1371/journal.pntd.0003139

**Published:** 2014-09-11

**Authors:** Poppy H. L. Lamberton, Narcis B. Kabatereine, David W. Oguttu, Alan Fenwick, Joanne P. Webster

**Affiliations:** 1 Department of Infectious Disease Epidemiology, Imperial College London, London, United Kingdom; 2 Schistosomiasis Control Initiative at Vector Control Division – Ministry of Health, Kampala, Uganda; Swiss Tropical and Public Health Institute, Switzerland

## Abstract

**Background:**

Two Kato-Katz thick smears (Kato-Katzs) from a single stool are currently recommended for diagnosing *Schistosoma mansoni* infections to map areas for intervention. This ‘gold standard’ has low sensitivity at low infection intensities. The urine point-of-care circulating cathodic antigen test (POC-CCA) is potentially more sensitive but how accurately they detect *S. mansoni* after repeated praziquantel treatments, their suitability for measuring drug efficacy and their correlation with egg counts remain to be fully understood. We compared the accuracies of one to six Kato-Katzs and one POC-CCA for the diagnosis of *S. mansoni* in primary-school children who have received zero to ten praziquantel treatments. We determined the impact each diagnostic approach may have on monitoring and evaluation (M&E) and drug-efficacy findings.

**Method/Principle Findings:**

In a high *S. mansoni* endemic area of Uganda, three days of consecutive stool samples were collected from primary school-aged children (six - 12 years) at five time-points in year one: baseline, one-week-post-, four-weeks-post-, six-months-post-, and six-months-one-week-post-praziquantel and three time-points in years two and three: pre-, one-week-post- and four-weeks-post-praziquantel-treatment/retreatment (n = 1065). Two Kato-Katzs were performed on each stool. In parallel, one urine sample was collected and a single POC-CCA evaluated per child at each time-point in year one (n = 367). At baseline, diagnosis by two Kato-Katzs (sensitivity = 98.6%) or one POC-CCA (sensitivity = 91.7%, specificity = 75.0%) accurately predicted *S. mansoni* infections. However, one year later, a minimum of three Kato-Katzs, and two years later, five Kato-Katzs were required for accurate diagnosis (sensitivity >90%) and drug-efficacy evaluation. The POC-CCA was as sensitive as six Kato-Katzs four-weeks-post and six-months-post-treatment, if trace readings were classified as positive.

**Conclusions/Significance:**

Six Kato-Katzs (two/stool from three stools) and/or one POC-CCA are required for M&E or drug-efficacy studies. Although unable to measure egg reduction rates, one POC-CCA appears to be more sensitive than six Kato-Katzs at four-weeks-post-praziquantel (drug efficacy) and six-months-post-praziquantel (M&E).

## Author Summary

Schistosomiasis is a parasitic disease infecting over 200 million people. It remains a major public health concern despite treatment of over 120 million people in sub-Saharan Africa alone. Accurate diagnostic methods are essential for monitoring drug efficacy and long-term control program success. The World Health Organization recommends two Kato-Katz thick smears (Kato-Katzs) from a single stool for *Schistosoma mansoni* diagnosis to map prevalence and areas for control interventions. Although highly specific, Kato-Katzs are thought to be insensitive at low egg counts. The recently refined urine point-of-care circulating cathodic antigen test (POC-CCA) has been proposed as a diagnostic alternative for mapping areas for interventions, and potentially for assessing drug efficacy. Over three years we assessed the accuracy of six Kato-Katzs and a single POC-CCA in detecting infections in Ugandan primary-school children at 11 time points with repeated praziquantel treatments. Our results demonstrate that two Kato-Katzs accurately detect *S. mansoni* infection pre-treatment, but at least three days of two Kato-Katzs per stool or one POC-CCA are required for annual monitoring and treatment evaluation and/or drug-efficacy studies. One POC-CCA may be more sensitive in measuring *S. mansoni* prevalence than six Kato-Katzs, but its accuracies for rigorous intensity measures are still to be proven.

## Introduction

Schistosomiasis remains a major public health concern despite praziquantel reaching over 30 million people in endemic areas in 2013 [1]. Goals to eliminate schistosomiasis by 2020 have been articulated by the World Health Organization's (WHO) ‘Roadmap for Neglected Tropical Disease (NTD) Implementation’ [2], and the London Declaration of the NTD Coalition [3]. Accurate diagnostic techniques, recently highlighted by Gomes and colleagues [4], are essential for monitoring and evaluation (M&E) of mass drug administration (MDA) programs at all stages [5]–[9], and particularly when considering elimination [2], [10], [11] and/or drug-resistance pharmacovigilance [12], [13].

The WHO recommends two Kato-Katz thick smears (Kato-Katzs) from a single stool [14] for *Schistosoma mansoni* diagnosis to determine prevalence to map areas for control interventions [15]. Kato-Katzs have assumed 100% specificity, but large inter- and intra-specimen variation [16]–[18] and low sensitivity for the detection of low intensity infections have been reported [19]–[21]. In Brazil, where M&E programs use only one Kato-Katz, *S. mansoni* prevalence has been significantly underestimated in low intensity regions [22], [23]. This may be associated with overestimated cure rates (CRs) and praziquantel efficacy [24], potentially missing drug resistance. Conversely, using one Kato-Katz can overestimate infection intensities [22]. These differences in sensitivity for prevalence versus infection intensity highlight complex interactions with egg reduction rates (ERRs) as true intensities decrease. Inter-day variation of excreted egg numbers post-treatment remains poorly understood. Two Kato-Katzs are commonly used for annual M&E of program impact and/or drug-efficacy studies, without being rigorously tested in these scenarios. Detailed analyses of the sensitivity and specificity of single and multiple Kato-Katzs following praziquantel treatment is urgently required.

It is not possible to directly measure *S. mansoni* adult worm numbers, due to their location in the mesenteric system, with eggs counted from Kato-Katzs used as a proxy for infection intensity. Immunodiagnostics for adult worm circulating cathodic antigens (CCA) or circulating anodic antigens (CAA) detect current infections and are potentially more sensitive for diagnosis of cases in low transmission areas [25]–[27]. Recent developments using innovative immunomagnetic separation with several target CCAs [28] and novel monoclonal antibody diagnostics for serum CCA [27] show high sensitivity even in low endemic areas. Serum CAA tests are more accurate than urine CAA tests [29], with advances made on a, not yet commercially available, point-of-care-CAA (POC-CAA) [30]. In contrast, urine CCA tests were more accurate than serum CCA tests [29]. Being easier to collect and more socially acceptable than stool or blood, urine POC-CCAs were developed for rapid, non-invasive diagnostics and proposed as alternatives to Kato-Katzs for *S. mansoni* prevalence mapping [31]–[37]. One urine POC-CCA is as sensitive as two Kato-Katzs for *S. mansoni* diagnosis for prevalence mapping [35] or two years post-treatment [36] and has now been used to map *S. mansoni* prevalence in low intensity areas in Uganda [37]. Major POC-CCA limitations are their ability to only semi-quantitatively measure infection intensity [32], [34], [38], inaccuracy for *S. haematobium* infection detection [39], [40], and not measuring soil-transmitted helminth (STH) infections. Intensity of infection measures are vital for control program M&E and drug-efficacy evaluation. Therefore knowledge on the ability of POC-CCAs to detect *S. mansoni* intensity reductions is essential [34]–[36], [41].

**Figure 1:**
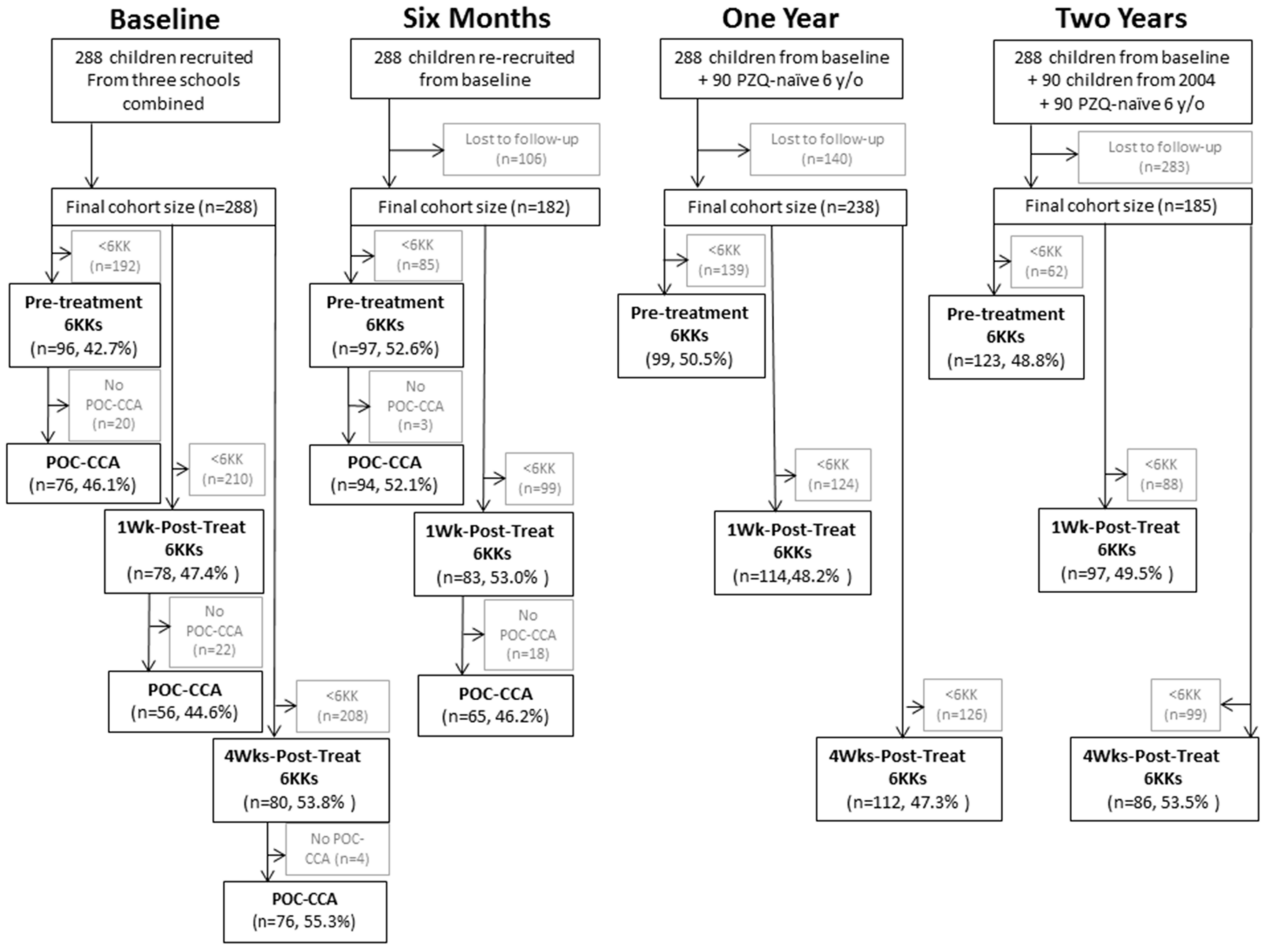
Recruitment and inclusion of primary-school children and their samples in the study. Final numbers of children who provided three stools for a total of six Kato-Katz thick smears (6KK) and one urine sample for a point-of-care circulating antigen test (POC-CCA) at each time-point. Final numbers and percentage female in parentheses. Totaling 1065 samples for Kato-Katz thick smears accuracy analyses and 367 for POC-CCA analyses over the whole study. Samples were collected pre-praziquantel treatment, one-week-post-praziquantel treatment (1 Wk-Post-Treat) and four-weeks-post-praziquantel treatment (4 Wks-Post-Treat) at four time points: Baseline, six months later, one year later and two years later.

We compared the accuracy of the currently available POC-CCA and one to six Kato-Katzs (two smears per day from three consecutive stool samples) for *S. mansoni* diagnosis, in primary-school children in Mayuge District, Uganda, at baseline and after up to ten praziquantel treatments per child over three years (STARD Checklist S1 and Figure 1). We evaluated the epidemiological implications of diagnostic methods for control program M&E and praziquantel-efficacy studies. We inform on the number of Kato-Katzs required to accurately detect *S. mansoni* infections pre- and post-praziquantel treatment and whether POC-CCAs are suitable alternatives. Our detailed longitudinal design enabled novel investigations into individuals' recent *versus* total praziquantel treatments, aiding biological understanding of differences between Kato-Katz and POC-CCA results post-praziquantel treatment. We predicted that the accuracy of M&E and drug-efficacy findings are limited by Kato-Katz sensitivity at low infection intensities post-treatment. We also predicted that a single POC-CCA would have a higher sensitivity than multiple Kato-Katzs, and be more informative for prevalence monitoring as control programs progress.

## Materials and Methods

### Ethics Statement

Approvals were granted by the Uganda National Council of Science and Technology (Memorandum of Understanding: sections 1.4, 1.5, 1.6) and the Imperial College Research Ethics Committee (EC NO: 03.36. R&D No: 03/SB/033E). Verbal assent was given by every child before inclusion into this study and at school committee meetings comprising of parents, teachers, and community leaders before the onset of the study. Written consent for the children to participate in the study was attained from each head teacher. Participation was voluntary and children could withdraw or be withdrawn from the study at any time without obligation. Children were treated with 40 mg/kg praziquantel and 400 mg albendazole (active against STH infections) as detailed below.

### Study Area

Samples were collected, between 2004 to 2006, from primary-school children, in a high *S. mansoni-*endemic area, in Mayuge district, Uganda from three schools on the shores of Lake Victoria: Bugoto Lake View, Bwondha, and Musubi Church of God. Children at Musubi were, to the authors' knowledge, praziquantel-naïve. Children at Bugoto and Bwondha had received 40 mg/kg praziquantel one year previously in 2003 [42]. Inclusion criteria were to have lived in the area since birth and to attend the schools sampled.

### Study Cohort and Treatment

In 2004, samples were collected at five time-points: baseline, one-week-post-, four-weeks-post-, six-months-post- and six-months-one-week-post-praziquantel treatment (Figure 1). In 2005 and 2006, samples were collected pre-, one-week-post-, and four-weeks-post-praziquantel re/treatment. On the third day of sampling, at baseline, six-months, one-year, and two-years all children were treated with 40 mg/kg praziquantel and 400 mg albendazole (active against STH infections). At one-week post-treatment, children with infections of >100 *S. mansoni* eggs per gram of stool (EPG) were retreated with 40 mg/kg praziquantel. At all other time-points all children with positive diagnoses for *S. mansoni* or STHs were retreated with 40 mg/kg praziquantel and 400 mg albendazole respectively.

Cohort and sample collection are described elsewhere [43]. In brief, 110 children from Bugoto, 110 from Bwondha and 68 from Musubi were recruited in 2004 with an equal sex ratio, aged six to 12 years, without prior knowledge of infection status and/or symptoms of *S. mansoni* infection. In addition, at one- and two-years, 30 praziquantel-naïve six year old children were recruited at each school and followed up with the original cohorts at the time points described above. This enabled monitoring of the impact of MDA on untreated children entering the school system, assessing diagnostic accuracies for Kato-Katzs and POC-CCA, in praziquantel-naïve and praziquantel-exposed children, as control programs progress.

### Intensity of Infection and Prevalence Measures

Diagnostic accuracy increases with the number of Kato-Katzs, however, in Brazilian low intensity regions, the additional benefit of more than six Kato-Katzs from repeated stools was negligible [21], supporting our six Kato-Katzs ‘gold standard’. Stool samples, marked with unique child IDs, were collected on three consecutive days, between 10:00 and 12:00 hours. Two 41.7 mg Kato-Katzs were prepared per stool and read onsite using a compound microscope with natural light source, by highly trained personnel from the Ugandan Vector Control Division, Ministry of Health. *S. mansoni*, hookworm, *Ascaris lumbricoides*, and *Trichuris trichiura* egg counts were recorded. Five percent of slides were reread after the study for *S. mansoni*, *A. lumbricoides*, and *T. trichiura* egg counts for quality control, but no significant differences were observed. One urine sample per child was collected between 10:00 and 12:00 hours on the first day. In the first year, at all five time-points, POC-CCAs (European Veterinary Laboratory, The Netherlands) were performed, according to the producer's protocols, by the first author, blind of other test results. Microhematuria was tested for using Hemastix (Bayer, United Kingdom).

### Statistical Analysis

SPSS version 19 (SPSS, Inc., Chicago, IL, United States of America) was used for all statistical analyses. The double entered data were not normally distributed and could not be normalized by transformation, therefore non-parametric tests were used. Individuals without the full six Kato-Katzs were excluded from the study (Figure 1). Arithmetic mean infection intensities were categorized as by the WHO (*S. mansoni*: light = 1–99 EPG, moderate = 100–399 EPG and high ≥400 EPG; *A. lumbricoides*: light = 1–4999 EPG; Hookworm: light = 1–1,999 EPG; *T. trichiura*: light = 1–999 EPG) [15]. Exact confidence intervals (CIs) were calculated for prevalence measures and standard errors for EPGs.

#### Inclusion bias and potential confounders

There were no significant differences between the final dataset (six Kato-Katzs) and the excluded dataset (<six Kato-Katzs) in *S. mansoni* intensities (Mann Whitney: U = 509672.5, d.f. = 1420, *p* = 0.12), STH presence (χ^2^ = 0.90, d.f. = 2, *p* = 0.34) or microhematuria (χ^2^ = 1.51, d.f. = 4, *p* = 0.83). It was therefore assumed that the final dataset was not biased by missing data. There were no significant differences in accuracies of POC-CCAs, with trace counted as negative (POC-CCA-t−) (Fisher's exact: P_A_ = 0.91, P_B_ = 0.91) or positive (POC-CCA-t+) (P_A_ = 0.93, P_B_ = 0.87) between those infected with STHs (n = 107) or not (n = 260). A greater proportion of the microhematuria positive samples (19/367, of which 17 were female) were POC-CCA negative than expected (χ^2^ = 17.62, d.f. = 2, *p*<0.001), although microhematuria was not thought to have biased results, with 12 negative, two trace, four positive (+) and one double positive (++) POC-CCA observed. All urine samples were screened for *S. haematobium* but no eggs were observed, confidently excluding *S. haematobium* co-infections.

#### Diagnostic accuracy of tests pre- and post-praziquantel-treatment

The sensitivity, specificity, positive predictive value (PPV) and negative predictive value (NPV) of POC-CCA-t+, POC-CCA-t−, and one to five Kato-Katzs were calculated with 95% exact CIs. Differences in sensitivity between our six Kato-Katzs ‘gold standard’ and the current recommended two Kato-Katzs or one POC-CCA were determined using the McNemar test. The agreement between six Kato-Katzs and two Kato-Katzs or POC-CCA were assessed using Kappa (κ) statistics: κ<0.01 no agreement; κ = 0.01–0.2 poor; κ = 0.21–0.4 fair; κ = 0.41–0.6 moderate; κ = 0.61–0.8 substantial; κ = 0.81–1 almost perfect [44]. Accuracies were also calculated for POC-CCA and one to six Kato-Katzs, using the combined ‘gold standard’ of POC-CCA-t+ and six Kato-Katzs [41]. Finally, the accuracies of one to five Kato-Katzs and POC-CCA were calculated over time since each individual had first ever been treated, to explore differences between community and individual treatment history on diagnostic accuracies.

#### Effect of mean infection intensity on accuracy

Data from each school, at each time-point, were used to compare sensitivities of one to five Kato-Katzs against school arithmetic mean EPG, using Spearman's rank correlation. Five best fit linear lines (for one to five Kato-Katzs) were calculated. The mean distance of data-points from these lines were compared, using Mann-Whitney, between two data-subsets to test whether the distance was greater for time-points one- or four-weeks-post-praziquantel in comparison to ‘pre-praziquantel’ (pre, six-months, one-year and two-years). No significant differences were found (all *p*>0.05) and data-points were combined for analysis of the effect of infection intensity.

#### Effect of diagnostic test on treatment efficacy and reinfection measures

Cure rates were determined, for each diagnostic method, as the proportion of *S. mansoni-*positive individuals at baseline who were negative four-weeks-post-praziquantel. Odds ratios were calculated for comparisons of prevalence at each time-point. Comparisons of infection intensities, over three days of Kato-Katzs, were performed using the Friedman test for repeated measures.

#### Ability of Kato-Katzs and POC-CCA to measure infection intensity pre- and post-praziquantel

Band strengths of the POC-CCAs (negative, trace, +, ++ and +++) were compared with Kato-Katz infection intensity categories and individual children's arithmetic mean EPG using Spearman's rank coefficient.

## Results

There were 1065 samples with six Kato-Katzs and 367 samples with six Kato-Katzs and a POC-CCA result (Figure 1). Baseline *S. mansoni* prevalence (χ^2^ = 0.38, d.f. = 2, *p* = 0.83) and EPG intensity (Kruskal-Wallis: H = 3.416, d.f. = 2, *p* = 0.18) were not significantly different between schools and all statistics were performed on the combined data. Baseline prevalence in the main (six Kato-Katzs) dataset was 94.8% with an arithmetic mean infection intensity of 249.8 EPG, similar to the POC-CCA dataset prevalence (94.7%) and intensity (259.0 EPG) (Table 1). Hookworm prevalence was 51.0%, whilst *A. lumbricoides* and *T. trichiura* infections were low at 1.0% and 9.4%, respectively (Table 1). *S. mansoni* prevalence at baseline, as recorded by POC-CCA-t+ and POC-CCA-t− was 88.2% and 78.9%, respectively (Table 1).

**Table 1:** Baseline prevalence and intensities.

						Infection intensity counts (%)		
Parasite	Diagnostic approach	Inclusion criteria	No. of children included	Prevalence % (95% CI)	Mean infection intensity (SE)	Light	Moderate	Heavy
*S. mansoni*	Six Kato-Katzs	Six Kato-Katzs	96	94.8 (88.3–98.3)	249.8 (30.2)	36 (39.6)	35 (38.5)	20 (22.0)
	Six Kato-Katzs	Six Kato-Katzs & one POC-CCA	76	94.7 (87.1–98.5)	259.0 (35.8)	28 (38.9)	27 (37.5)	17 (23.6)
	POC-CCA-t−	Six Kato-Katzs & one POC-CCA	76	78.9 (68.1–87.5)	na	24 (40.0)	33 (55.0)	3 (5.0)
	POC-CCA-t+	Six Kato-Katzs & one POC-CCA	76	88.2 (78.7–94.4)	na	31 (46.3)	33 (49.3)	3 (5.4)
	Six Kato-Katzs & one POC-CCA-t+	Six Kato-Katzs & one POC-CCA	76	94.7 (87.1–98.5)	na	na	na	na
*A. lumbricoides*	Six Kato-Katzs	Six Kato-Katzs	96	1.0 (0.0–5.7)	1 (1)	1 (100)	0	0
Hookworm	Six Kato-Katzs	Six Kato-Katzs	96	51.0 (40.6–61.4)	104.3 (18.4)	49 (100)	0	0
*T. trichiura*	Six Kato-Katzs	Six Kato-Katzs	96	9.4 (4.4–17.1)	6.1 (2.4)	9 (100)	0	0

Overall baseline prevalence (measured by six Kato-Katz thick smears (Kato-Katzs) and/or one rapid urine based point-of-care circulating cathodic antigen test (POC-CCA) if trace readings are counted as positive (POC-CCA-t+) or negative (POC-CCA-t−)) and intensities (arithmetic mean eggs per gram of stool) of helminth infections in three primary schools in Mayuge District, Uganda. CI = confidence interval, SE = standard error, na = not applicable. Infection intensity categories are as per World Health Organization guidelines [15].

### Diagnostic Accuracies Pre- and Post-Praziquantel-Treatment

#### Kato-Katz

**Figure 2:**
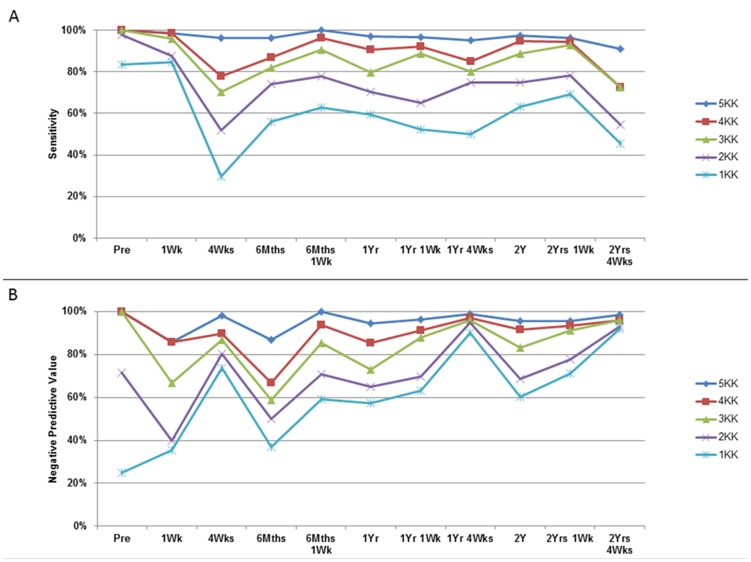
Sensitivity and negative predictive values of one to five Kato-Katzs for *S. mansoni* diagnosis. (A) Sensitivity and (B) Negative Predictive Values of one to five Kato-Katzs thick smears (1KK to 5KK) for *S. mansoni* diagnosis over 11 time-points using six Kato-Katzs as the ‘gold standard’. All individuals were treated with 40 mg/kg praziquantel after the start of the study (pre) and then again at six-months, one-year and two-years. All individuals providing stool samples with >100 eggs per gram (EPG) at one-week-post-praziquantel treatment and any infected children at all other time-points were re-treated with 40 mg/kg praziquantel. 95% confidence intervals are excluded for clarity, but can be seen in Table 2.

There were no significant differences between the sensitivity and NPV of one to five Kato-Katzs between the POC-CCA and Kato-Katzs only datasets (all *p*>0.05) (Figure 2, Tables 2 and 3). At baseline, 97% of *S. mansoni* infections were detected by two Kato-Katzs, reaching 100% with a second day of sampling (Figure 2). At one-week-post-praziquantel, sensitivity of two Kato-Katzs was approximately 90%. However, by four-weeks-post-praziquantel, nearly half of infected individuals were wrongly classified as uninfected with the sensitivity of two Kato-Katzs being only 51.9%. Four Kato-Katzs had a higher sensitivity of 77.8%, but five Kato-Katzs were required for sensitivities above 90% at four-weeks-post-praziquantel. The sensitivity of two Kato-Katzs at six-month-post-praziquantel was 74.0%, but five Kato-Katzs were required to reach sensitivities above 90%. As praziquantel treatments continued, the sensitivities of one to five Kato-Katzs remained systematically lower than at baseline (Figure 2A, Tables 2 and 3).

**Table 2:** The accuracy of one to five Kato-Katzs for detecting *S. mansoni* infection.

	Baseline			One Week			Four Weeks			Six Months			Six Months One Week		
	Prev = 94.8% (88.3–98.3)			92.3% (84.0–97.1)			33.8% (23.6–45.2)			79.4% (70.0–86.9)			65% (53.8–75.2)		
Kato-Katz	Sens	Spec	NPV	Sens	Spec	NPV	Sens	Spec	NPV	Sens	Spec	NPV	Sens	Spec	NPV
**One**	83.50%	100%	25%	84.70%	100%	35.30%	29.60%	100%	73.60%	55.80%	100%	37.00%	63.00%	100%	59.20%
	(74.3–90.5)	(47.8–100)	(8.7–49.1)	(74.3–92.1)	(54.1–100)	(14.2–61.7)	(13.8–50.2)	(93.3–100)	(61.9–83.3)	(44.1–67.2)	(83.2–100)	(24.3–51.3)	(48.7–75.7)	(88.1–100)	(44.2–73.0)
**Two**	97.80%	‘’	71.40%	87.50%	‘’	40%	51.90%	‘’	80.30%	74.00%	‘’	50.00%	77.80%	‘’	70.70%
	(92.3–99.7)		(29.0–96.3)	(77.6–94.1)		(16.3–67.7)	(31.9–71.3)		(68.7–89.1)	(62.8–83.4)		(33.8–66.2)	(64.4–88.0)		(54.5–83.9)
**Three**	100%	‘’	100%	95.80%	‘’	66.70%	70.40%	‘’	86.90%	81.80%	‘’	58.80%	90.70%	‘’	85.30%
	(96.0–100)		(47.8–100)	(88.3–99.1)		(29.9–92.5)	(49.8–86.2)		(75.8–94.2)	(71.4–89.7)		(40.7–75.4)	(79.7–96.9)		(68.9–95.0)
**Four**	100%	‘’	100%	98.60%	‘’	85.70%	77.80%	‘’	89.80%	87.00%	‘’	66.70%	96.30%	‘’	93.50%
	(96.0–100)		(47.8–100)	(92.5–100)		(42.1–99.6)	(57.7–91.4)		(79.2–96.2)	(77.4–93.6)		(47.2–82.7)	(87.3–99.5)		(78.6–99.2)
**Five**	100%	‘’	100%	98.60%	‘’	85.70%	96.30%	‘’	98.10%	98.70%	‘’	95.20%	100%	‘’	100%
	(96.0–100)		(47.8–100)	(92.5–100)		(42.1–99.6)	(81.0–99.9)		(90.1–100)	(93.0–100)		(76.2–99.9)	(93.4–100)		(88.1–100)

	One Year			One Year One Week			One Year Four Weeks		
	Prev = 68.1% (57.7–77.3)			55.3% (45.7–64.6)			17.9% (11.3–26.2)		
	Sens	Spec	NPV	Sens	Spec	NPV	Sens	Spec	NPV
**One**	59.40%	100%	57.40%	50.80%	100%	62.20%	50.00%	100%	90.20%
	(46.4–71.5)	(90.0–100)	(44.1–70.0)	(37.9–63.6)	(93.0–100)	(50.8–72.7)	(27.2–72.8)	(96.1–100)	(82.7–95.2)
**Two**	70.30%	‘’	64.80%	63.50%	‘’	68.90%	60.00%	‘’	92.00%
	(57.6–81.1)		(50.6–77.3)	(50.4–75.2)		(57.1–79.2)	(36.1–80.9)		(84.8–96.5)
**Three**	79.70%	‘’	72.90%	87.30%	‘’	86.40%	80.00%	‘’	95.80%
	(67.8–88.7)		(58.2–84.7)	(76.5–94.4)		(75.0–94.0)	(56.3–94.3)		(89.7–98.9)
**Four**	90.60%	‘’	85.40%	90.50%	‘’	89.50%	80.00%	‘’	95.80%
	(80.7–96.5)		(70.8–94.4)	(80.4–96.4)		(78.5–96.0)	(56.3–94.3)		(89.7–98.9)
**Five**	96.90%	‘’	94.60%	96.80%	‘’	96.20%	95.00%	‘’	98.90%
	(89.2–99.6)		(81.8–99.3)	(89.0–99.6)		(87.0–99.5)	(75.1–99.9)		(94.2–99.9)

	Two Years			Two Years One Week			Two Years Four Weeks		
	Prev = 64.2% (55.1–72.7)			56.7% (46.3–66.7)			12.8% (6.6–21.7)		
	Sens	Spec	NPV	Sens	Spec	NPV	Sens	Spec	NPV
**One**	60.30%	100%	60.30%	69.10%	100%	71.20%	45.50%	100%	92.80%
	(51.7–73.9)	(92.0–100)	(48.1–71.5)	(55.2–80.9)	(91.6–100)	(57.9–82.2)	(16.7–76.6)	(95.2–100)	(84.6–97.2)
**Two**	68.80%	‘’	68.80%	78.20%	‘’	77.80%	54.50%	‘’	93.80%
	(63.6–83.8)		(55.9–79.8)	(65.0–88.2)		(64.4–88.0)	(23.4–83.3)		(86.0–97.9)
**Three**	83.00%	‘’	83.00%	92.70%	‘’	91.10%	72.70%	‘’	96.20%
	(79.5–94.7)		(70.2–91.9)	(82.4–98.0)		(79.2–97.6)	(39.0–94.0)		(89.2–99.2)
**Four**	91.70%	‘’	91.70%	94.50%	‘’	93.30%	72.70%	‘’	96.20%
	(87.5–98.6)		(80.0–97.7)	(84.9–98.9)		(81.7–98.6)	(39.0–94.0)		(89.2–99.2)
**Five**	95.70%	‘’	95.70%	96.40%	‘’	95.50%	100%	‘’	100%
	(91.2–99.7)		(85.2–99.5)	(87.5–99.6)		(84.5–99.4)	(71.5–100)		(95.2–100)

The accuracy of one to five Kato-Katz thick smears for detecting *S. mansoni* infection with prevalence reported for diagnosis using six Kato-Katz thick smears as the ‘gold standard’. There were 11 time-points where children were sampled over three years, from the complete dataset of children who each had six Kato-Katz thick smear readings (n = 1065). 95% confidence intervals are shown in parentheses. Prev = prevalence, Sens = sensitivity, Spec = specificity, NPV = negative predictive value.

**Table 3:** The accuracy of one to six Kato-Katzs and one point-of-care circulating cathodic antigen test for diagnosing *S. mansoni* infections.

	Baseline (n = 76)				One Week (n = 56)				Four Weeks (n = 76)				Six Months (n = 94)				Six Months One Week (n = 65)			
	Prev = 94.7% (87.2–97.9)				91.1% (80.7–96.1)				34.2% (24.5–45.4)				79.8% (70.6–86.7)				69.2% (57.2–79.1)			
Gold Standard	Intensity = 259.0 EPG (187.7–330.3)				213.9 EPG (139.7–288.1)				8.3 EPG (1.5–15.1)				41.9 EPG (22.9–60.9)				42.7 EPG (24.4–61.0)			
6 Kato-Katzs	Sens	Spec	NPV	PPV	Sens	Spec	NPV	PPV	Sens	Spec	NPV	PPV	Sens	Spec	NPV	PPV	Sens	Spec	NPV	PPV
**1 Kato-Katz**	84.7%	100.0%	26.7%		90.2%	100.0%	45.5%		30.8%	100.0%	73.5%		57.3%	100%	37.3%		66.7%	100%	57.1%	
	(74.7–91.3)	(51.0–100)	(10.9–52.0)		(79.0–95.7)	(56.6–100)	(21.3–72.0)		16.5–50.0)	(92.9–100)	(62.0–82.6)		(46.1–67.9)	(83.9–100)	(24.1–51.9)		52.1–78.6)	3.9–100)	(40.9–72.0)	
**2 Kato-Katzs**	98.6%	“	80.0%		92.2%	“	55.6%		50.0%	“	79.4%		73.3%	“	48.7%		80.0%	“	69.0%	
	(92.5–99.8)		(37.6–96.4)		(81.5–96.9)		(26.7–81.1)		32.1–67.9)		(67.6–87.5)		(62.4–82.0)		(32.4–65.2)		66.2–89.1)		(50.8–82.7)	
**3 Kato-Katzs**	100%	“	100%		98.0%	“	83.3%		69.2%	“	86.2%		81.3%	“	57.6%		91.1%	“	83.3%	
	(94.9–100)		(51.0–100)		(89.7–99.7)		(43.7–97.0)		(50.0–83.5)		(75.1–92.9)		(71.1–88.5)		(39.2–74.5)		(79.3–96.5)		(64.2–93.3)	
**4 Kato-Katz**	100%	“	100%		100%	“	100%		76.9%	“	89.3%		86.7%	“	65.5%		95.6%	“	90.9%	
	(94.9–100)		(51.0–100)		(93.0–100)		(56.6–100)		(58.0–89.0)		(78.5–95.0)		(77.2–92.6)		(45.7–82.1)		(85.2–98.8)		(72.2–97.5)	
**5 Kato-Katz**	100%	“	100%		100%	“	100%		96.2%	“	98.0%		98.7%	“	95.0%		100%	“	100%	
	(94.9–100)		(51.0–100)		(93.0–100)		(56.6–100)		(81.1–99.3)		(89.7–99.7)		(92.8–99.8)		(75.1–99.9)		(92.1–100)		(83.9–100)	
**POC-CCA-t−**	83.3%	100%	25.0%	100%	27.5%	100%	11.9%	100%	61.5%	82.0%	80.4%	64.0%	92.0%	5.3%	14.3%	79.3%	64.4%	75.0%	48.4%	85.3%
	(73.1–90.2)	(51.0–100)	(10.2–49.5)	(94.0–100)	(17.1–41.0)	(56.6–100)	(5.2–25.0)	(93.0–100)	(42.5–77.6)	(69.2–90.0)	(67.5–89.0)	(44.5–79.8)	(83.6–96.3)	(0.9–24.6)	(2.6–51.3)	(69.7–86.5)	(49.8–76.8)	(53.1–88.9)	(32.0–65.2)	(69.9–93.6)
**POC-CCA-t+**	91.7%	75.0%	33.3%	98.5%	66.7%	100%	22.7%	100%	73.0%	58.0%	80.6%	47.5%	92.0%	5.3%	14.3%	79.3%	7560.0%	65.0%	54.2%	82.9%
	(83.0–96.1)	(30.1–95.4)	(12.1–64.6)	(92.0–99.7)	(53.0–78.0)	(56.6–100)	(10.1–43.4)	(93.0–100)	(53.9–86.3)	(58.0–70.6)	(65.0–90.3)	(32.9–62.5)	(83.4–97.0)	(0.9–24.6)	(2.6–51.3)	(69.7–86.5)	(61.3–85.8)	(43.3–81.9)	(35.1–72.1)	(68.7–91.5)

	Baseline (n = 76)				One Week (n = 56)				Four Weeks (n = 76)				Six Months (n = 94)				Six Months One Week (n = 65)			
	Prev = 96.1% (88.9–99.2)				91.1% (80.4–97.0)				61.8% (50.0–72.8)				98.9% (94.2–100)				80.0% (68.2–88.9)			
6 Kato-Katzs & POC-CCA-t+	Sens	Spec	NPV	PPV	Sens	Spec	NPV	PPV	Sens	Spec	NPV	PPV	Sens	Spec	NPV	PPV	Sens	Spec	NPV	PPV
**1 Kato-Katz**	83.6%	100%	20.0%		90.2%	100%	45.5%		17.0%	100%	42.6%		46.2%	100%	2.0%		57.7%	100%	37.1%	
	(73.4–90.3)		(7.1–45.2)		(79.0–95.7)	(56.6–100)	(21.3–72.0)		(8.9–30.1)	(88.3–100)	(31.6–54.5)		(36.5–56.3)	(20.7–100)	(0.4–10.3)		(44.2–70.1)	(77.2–100)	(23.2–53.7)	
**2 Kato-Katzs**	97.3%	“	60.0%		92.2%	“	55.6%		27.7%	“	46.0%		59.1%	“	2.6%		69.2%	“	44.8%	
	(90.6–99.3)		(23.1–88.2)		(81.5–96.9)		(26.7–81.1)		(16.9–41.8)		(34.3–58.2)		(49.0–68.6)		(0.5–13.2)		(55.7–80.1)		(28.4–62.5)	
**3 Kato-Katzs**	98.6%	“	75.0%		98.0%	“	83.3%		38.3%	“	50.0%		65.6%	“	3.0%		78.8%	“	54.2%	
	(92.6–99.8)		(30.1–95.4)		(89.7–99.7)		(43.7–97.0)		(25.8–52.6)		(37.5–62.5)		(55.5–74.5)		(0.5–15.3)		(66.0–87.8)		(35.1–72.1)	
**4 Kato-Katz**	98.6%	“	75.0%		100%	“	100%		42.6%	“	51.8%		69.9%	“	3.4%		82.7%	“	59.1%	
	(92.6–99.8)		(30.1–95.4)		(93.0–100)		(56.6–100)		(29.5–56.7)		(39.0–64.3)		(59.9–78.3)		(0.6–17.2)		(70.3–90.6)		(38.7–76.7)	
**5 Kato-Katz**	98.6%	“	75.0%		100%	“	100%		53.2%	“	56.9%		79.6%	“	5.0%		86.5%	“	65.0%	
	(92.6–99.8)		(30.1–95.4)		(93.0–100)		(56.6–100)		(39.3–66.7)		(43.3–69.5)		(70.3–86.5)		(0.9–23.6)		(74.7–93.3)		(43.3–81.9)	
**5 Kato-Katz**	98.6%	“	75.0%		100%	“	100%		55.3%	“	58.0%		80.6%	“	5.3%		86.5%	“	65.0%	
	(92.6–99.8)		(30.1–95.4)		(93.0–100)		(56.6–100)		(41.3–68.6)		(44.2–70.6)		(71.5–87.4)		(0.9–24.6)		(74.7–93.3)		(43.3–81.9)	
**POC-CCA-t−**	82.2%	“	17.7%		27.5%	“	11.9%		53.2%	“	56.9%		93.5%	“	14.3%		65.4%	“	41.9%	
	(71.9–89.3)		(6.2–41.0)		(17.1–41.0)		(5.2–25.0)		(39.3–66.7)		(43.3–69.5)		(86.6–97.0)		(2.6–51.3)		(51.2–76.9)		(26.4–59.2)	
**POC-CCA-t+**	90.9%	“	33.3%		66.7%	“	22.7%		85.1%	“	80.6%		93.5%	“	14.3%		78.8%	“	54.2%	
	(81.6–95.8)		(12.1–64.9)		(53.0–78.0)		(10.1–43.4)		(85.1–92.6)		(65.0–90.3)		(86.6–97.0)		(2.6–51.3)		(66.0–87.8)		(35.1–72.1)	

One to six Kato-Katz thick smears (Kato-Katzs) and a single point-of-care circulating cathodic antigen test (POC-CCA) (comparing if trace readings are counted as positive (POC-CCA-t+) or negative (POC-CCA-t−)) for detecting *S. mansoni* infections in primary-school children in Mayuge District, Uganda, comparing with either a ‘gold standard’ of six Kato-Katzs (upper half of the table) or with both six Kato-Katzs and one POC-CCA (lower half of the table) for diagnosis. Sample sizes are included in the timeline on the top row, with prevalence (as measured by the respective ‘gold standard’) and intensity from six Kato-Katzs. NB prevalence and mean infection intensities differ from Table 2 and Figures 4A and 5 as these, here, are calculated from individuals with six Kato-Katzs and POC-CCA results (n = 367), rather than just six Kato-Katzs (n = 1065) (Figure 1). 95% confidence intervals are shown in parentheses. Prev = prevalence, Sens = sensitivity, Spec = specificity, NPV = negative predictive value, PPV = positive predictive value.

At baseline, only a quarter of the individuals found to be negative by one Kato-Katz were negative for six Kato-Katzs, with three Kato-Katzs required for a NPV of 100% (Figure 2B, Tables 2 and 3). In each year of the study, NPVs peaked four weeks after treatment, but were at their lowest immediately before the next round of treatment (Figure 2B, Table 2).

**Figure 3:**
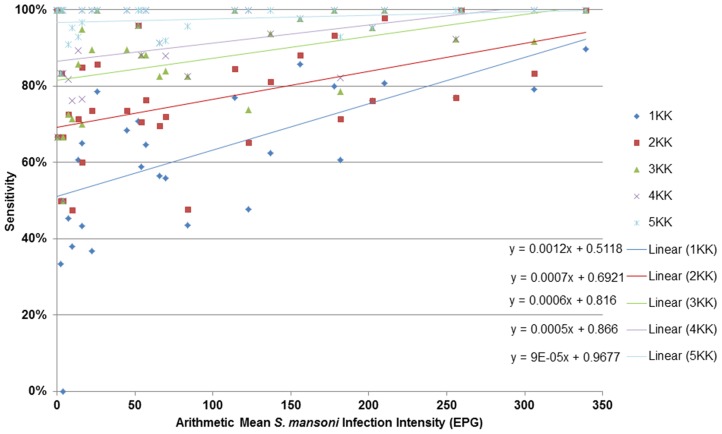
Effect of *S. mansoni* arithmetic mean infection intensity on sensitivity of one to five Kato-Katzs. The sensitivity of one to five Kato-Katz thick smears (1KK to 5KK) for diagnosing *S. mansoni* infections at a range of community arithmetic mean *S. mansoni* infection intensities (measured as eggs per gram of stool (EPG) from six Kato-Katz thick smears). *S. mansoni* infection intensities were measured at three primary schools, at 11 time points each, ranging from pre-treatment to two-years-four-weeks-post-praziquantel treatment. Lines are best fit linear lines.

The sensitivity of one to four Kato-Katzs was positively correlated with the mean infection intensity for one Kato-Katz (r = 0.539, *p* = 0.001); two Kato-Katzs (r = 0.427, *p* = 0.02); three Kato-Katzs (r = 0.424, *p* = 0.02); four Kato-Katzs (r = 0.384, *p* = 0.03), but not five Kato-Katzs (r = 0.125, *p* = 0.50) (Figure 3). Best fit lines indicated that in a school with an arithmetic mean infection intensity of, for example 300 EPG, two Kato-Katzs had 90% sensitivity, whereas in a school with an arithmetic mean infection intensity of 100 EPG, at least four Kato-Katzs were required for 90% sensitivity (Figure 3).

At baseline, the agreement between two Kato-Katzs and six Kato-Katzs was almost perfect (κ = 0.82), however at all other times the agreements were only substantial or moderate. The sensitivity of two Kato-Katzs was not significantly different to the ‘gold standard’ (*p* = 0.15) at baseline, but was significantly lower at all other times (*p*<0.001 to 0.03).

#### POC-CCA

POC-CCA-t+ had a high sensitivity at baseline of 91.7%, but a low sensitivity at one- and four-weeks-post-praziquantel of 66.7% and 73.0% respectively (Table 3). Sensitivity returned to baseline levels by six-months-post-praziquantel at 92.0% (Table 3). POC-CCA specificity varied with time since praziquantel (Table 3). Only one of the 19 children classified as negative by six Kato-Katzs had a negative POC-CCA result six-months-post-praziquantel. All POC-CCA-t− and -t+ PPVs were ≥80% except at four-weeks-post-praziquantel. Conversely, the NPVs were continuously low except at four-weeks-post-praziquantel. POC-CCA-t+ showed a moderate agreement with the ‘gold standard’ at baseline (κ = 0.42), but at all other time-points, only poor or no agreement. POC-CCA-t− showed a fair agreement at baseline and six-months-one-week (κ = 0.34), but a moderate agreement at four-weeks (κ = 0.44). POC-CCA-t+ results were significantly different from six Kato-Katzs at one-week, four-weeks and six-months (all *p*≤0.01). POC-CCA-t− results were significantly different from six Kato-Katzs at all time-points except four-weeks-post-praziquantel (all *p*≤0.02).

### Effect of Diagnostic Test on Treatment Efficacy and Reinfection

**Figure 4:**
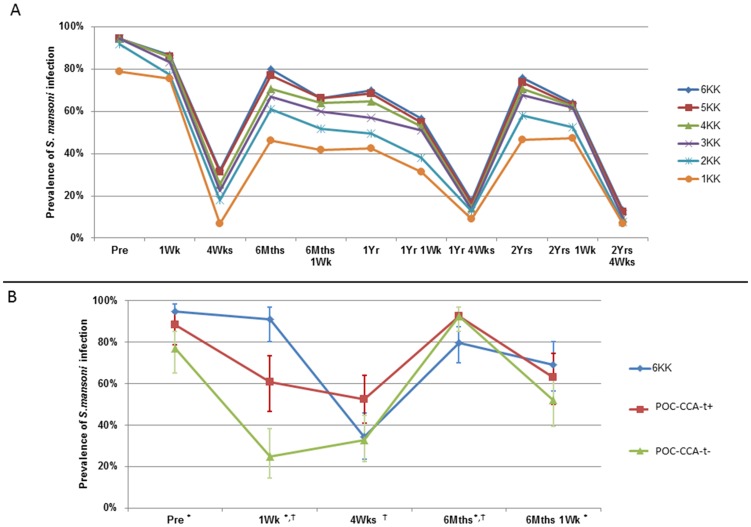
Effect of diagnostic technique and sampling effort on *S. mansoni* prevalence measures. The effect of (A) number of Kato-Katz thick smears (Kato-Katzs), from one to six (1KK to 6KK) on the recorded prevalence of *S. mansoni* infection with multiple praziquantel treatments. All individuals were treated after sampling at pre, six-months, one-year and two-years. All individuals excreting more than 100 EPG at one-week and any infected children at all other time-points were re-treated. 95% confidence intervals are excluded for clarity. Difference in sensitivities between the current standard two Kato-Katzs and our ‘gold standard’ of six Kato-Katzs determined by the McNemar test is significant at all time-points except baseline. The effect of (B) diagnosis method using six Kato-Katzs or one point-of-care circulating cathodic antigen test (POC-CCA), with a trace counting as either a positive (POC-CCA-t+) or negative (POC-CCA-t−) on *S. mansoni* infection prevalence with 95% confidence intervals. Difference in sensitivities between the POC-CCA and our ‘gold standard’ of six Kato-Katzs determined by the McNemar test: ^*^ significant for POC-CCA-t− or ^†^ significant POC-CCA-t+.

**Figure 5:**
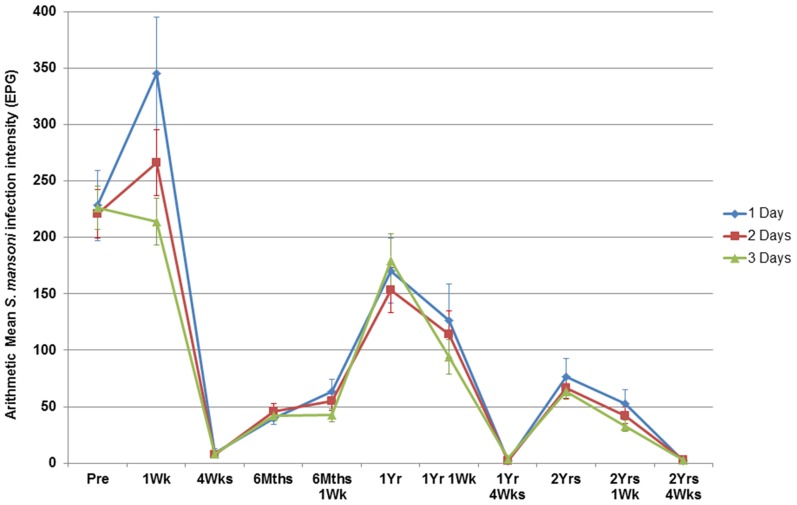
*S. mansoni* intensity measures from one to three days of duplicate Kato-Katz thick smears. Data shown over multiple rounds of praziquantel treatments with standard error bars.

Observed cure (measured at four-weeks-post-praziquantel treatment) and reinfection (measured at six-months) rates depended on sampling method and effort (Figures 4 and 5). Two Kato-Katzs underestimated *S. mansoni* reinfection whilst overestimating CRs (Figure 4A) (two Kato-Katzs CR = 81.5%; six Kato-Katzs CR = 70.4%). Cure rates determined with POC-CCA-t− were 47.8% and 26.1% for POC-CCA-t+. One-week-post-recent-praziquantel (from data at both one-week and six-months-one-week), prevalence was significantly lower when measured by POC-CCAs than by six Kato-Katzs (Figure 4B) (OR 0.33 (95% CI: 0.19, 0.59).

Pre-re/treatment and at four-weeks-post-praziquantel-re/treatment in years zero, one, and two, the number of days of Kato-Katzs did not significantly affect the infection intensities (Figure 5) (all p>0.05). However, at one-week-post-re/treatment (one-week, six-months-one-week, one-year-one-week and two-years-one-week), two Kato-Katzs and to a lesser extent four Kato-Katzs systematically, and significantly, overestimated the mean infection intensity (Friedman χ^2^ = 33.08, d.f. = 2, *p*<0.001).

**Figure 6:**
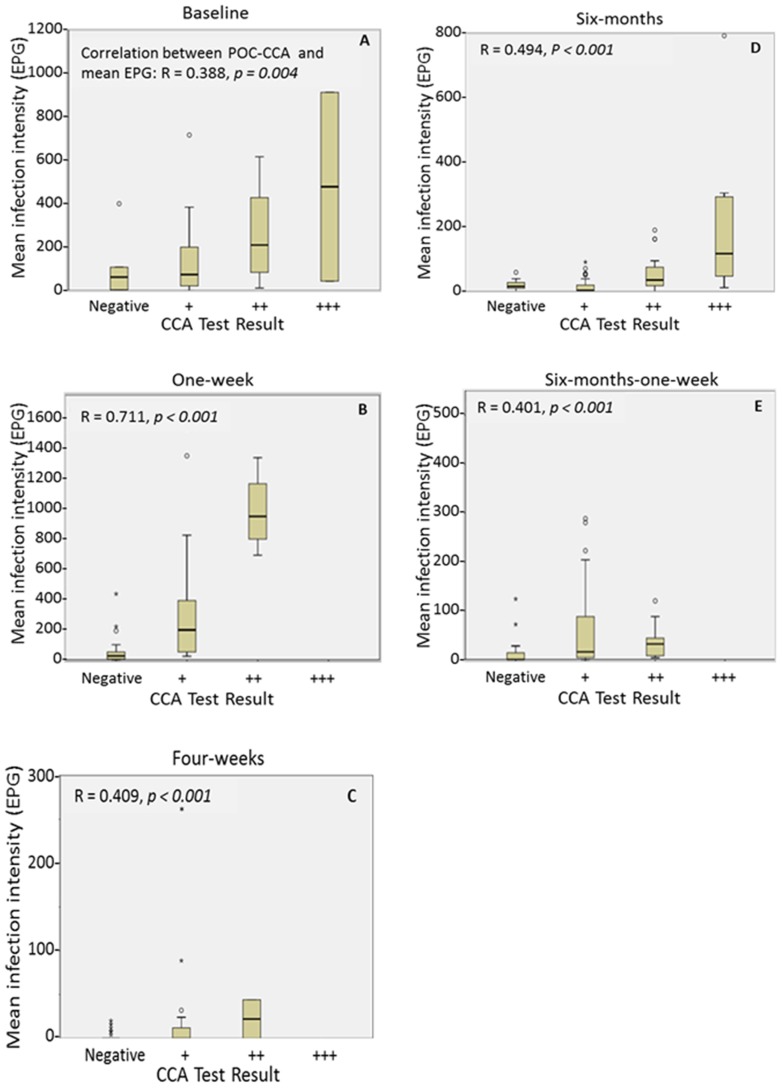
Comparison of *S. mansoni* arithmetic mean intensity measured by six Kato-Katzs with POC-CCA band strength. *S. mansoni* mean intensity in eggs per gram of stool (EPG) in comparison to the band strengths of a single urine point-of-care circulating cathodic antigen test (CCA) at five time-points, from (A) pre-treatment at baseline, (B) one-week-post-, (C) four-weeks-post-, (D) six-months-post- and (E) six-months-one-week-post-praziquantel treatment. Spearman's rank coefficients and significance values are given for each time point. Trace readings were included in the + category. NB y axes vary.

Infection intensity measured by the strength of the POC-CCA bands (graded from negative, + (inc. trace), ++ and +++) did not accurately predict the six Kato-Katzs infection intensity categories (negative, light, moderate, and heavy) (Table S1). However, strong positive correlations were seen between the ordinal POC-CCA band strengths and Kato-Katzs infection intensity categories (baseline: r = 0.402, *p* = 0.003; one-week: r = 0.647, *p*<0.001; four-weeks: r = 0.389, *p* = 0.001; six-months: r = 0.413, *p*<0.001; six-months-one-week: r = 0.424, *p*<0.001) as well as between POC-CCA band strengths and individual arithmetic mean EPG (Figures 6A to E).

### Six Kato-Katzs and POC-CCA as the ‘Gold Standard’

Pre-treatment, one POC-CCA-t+, and two Kato-Katzs had sensitivities above 90%, however the POC-CCA-t− and t+ NPVs were extremely low, similar to one Kato-Katz (Table 3). At one-week-post-praziquantel, one POC-CCA was less sensitive and had lower NPVs than one Kato-Katz, whilst two Kato-Katzs had an NPV of only 55.6%. At four-weeks-post-praziquantel POC-CCA-t+ had high sensitivity and NPV (>80%), whilst POC-CCA-t− and one to six Kato-Katzs all had sensitivities and NPVs of <60%. Indeed, two Kato-Katzs only detected a quarter of the infections. At six-months-post-praziquantel POC-CCA-t+ was more sensitive than six Kato-Katzs, however all diagnostics showed NPV values below 15%.

### Effect of Individual Child's Praziquantel Treatment on Test Accuracies

Some Bugoto and Bwondha children had been treated once before the study and new praziquantel-naïve cohorts were recruited each year. We therefore also analyzed our data along timelines specific for each individual child's praziquantel exposure (Tables S2 and S3). Key results were not affected by re-analyzing the data in this manner. Pre-treatment, six-months, one-year and one-year-six-months POC-CCA-t+s showed high sensitivities but low NPVs throughout (Table S2). One-week-post-recent-praziquantel, three Kato-Katzs were required for >90% sensitivity in general, and four-weeks-post-recent-praziquantel four or five Kato-Katzs were required for >90% sensitivities (Table S3). An increased Kato-Katz sampling effort was required year on year to achieve sensitivities of >90% (Table S3), which was not clearly seen in the original M&E timeline (Figure 2, Table 2). Praziquantel-naïve children and children one-year-post-praziquantel required three Kato-Katzs for accurate *S. mansoni* diagnosis, whilst four Kato-Katzs were required at two-years, and five Kato-Katzs at three-years.

## Discussion

We evaluated one to six Kato-Katzs and one POC-CCA for *S. mansoni* diagnosis before and after multiple rounds of praziquantel treatment, and how test choice affects M&E and drug-efficacy interpretations. Our data support using one POC-CCA-t+ or two Kato-Katzs for pre-treatment mapping in high endemicity areas [35]–[37]. However, as MDA continues, five Kato-Katzs were required for diagnosis of children after three to ten praziquantel treatments, particularly pertinent with the Ugandan control program already in its 11^th^ year. The sensitivity of two Kato-Katzs was low four-weeks-post-praziquantel treatment indicating that, if using this method, half the individuals would erroneously indicate a cured/negative infection. The high sensitivity of two Kato-Katzs and their agreement with the ‘gold standard’ observed at baseline was not maintained over time and with recurring praziquantel treatments. Indeed, such high sensitivity and agreement was not observed again throughout this study.

POC-CCAs are shown to be more sensitive but less specific than two Kato-Katzs [33], [37], [45], [46]. Our data show that one POC-CCA-t+ at four-weeks-post-praziquantel for praziquantel-efficacy studies and six-months-post-praziquantel for M&E, was more sensitive than six Kato-Katzs at the same time periods. Our POC-CCA-t+ baseline sensitivity (91.7%), from a 94.8% *S. mansoni* prevalence population, was comparable with that previously published from Côte d'Ivoire (sensitivity = 86.9%, prevalence = 91.8%) [33]. In contrast, our 73% sensitivity at four-weeks-post-praziquantel (prevalence = 34.2%) was greater than in the low prevalence Côte d'Ivoire region (sensitivity = 56.3%, prevalence = 32.9%) [33]. This may be explained by that study's rigorous nine Kato-Katzs ‘gold standard’, with our six Kato-Katzs possibly still missing infections. In addition, in Côte d'Ivoire, three POC-CCAs were performed, increasing sensitivity, in comparison with our single POC-CCA [33]. The lack of POC-CCA reproducibility data, even from single urine samples [35], are a key limitation of our study. Though utilizing matching components, our accuracies from European Veterinary Laboratory POC-CCAs, may vary from the Rapid Medical Diagnostics' POC-CCAs used in Côte d'Ivoire, however differences were not observed at higher prevalence.

At four-weeks-post-praziquantel, prevalence levels as indicated by six Kato-Katzs and one POC-CCA was nearly double (61.8%) than for just six Kato-Katzs (34.2%). Cure rates using two Kato-Katzs were >80% *versus* 70% with six Kato-Katzs, and only ∼25% with POC-CCA-t+. Similar results have been seen for *S. haematobium* [47]. Further discordance between Kato-Katzs and POC-CCA at six-months (specificity of 5.3%) may be explained by high numbers of infections missed by Kato-Katzs. It is unlikely that POC-CCA false positives are the full explanation due to high specificity at four-weeks-post-praziquantel treatment, with potentially more ‘true’ negatives and only 1% of POC-CCA giving false positives in non-endemic areas [35]. We believe that the low POC-CCA specificities are, in part, due to low sensitivities of Kato-Katzs.

When six Kato-Katzs and one POC-CCA were the combined ‘gold standard’, baseline and one-week accuracies were relatively unaffected. Four-weeks-post-praziquantel Kato-Katzs sensitivities were substantially lower, having profound implications on what is a suitable ‘gold standard’ when communities have received multiple praziquantel treatments. Latent class modeling [35], [36], with additional diagnostics [39] overcome theoretical difficulties, but is not fully applicable in praziquantel-efficacy studies, providing weighted prevalence rather than individual infection and clearance data.

Studies from the same region in Uganda demonstrating reduced *S. mansoni* infection prevalence and intensity levels in response to MDA [42], [48], used only two Kato-Katzs and may have overestimated annual reductions [37]. We strongly recommend, as treatment campaigns continue, increased sampling efforts and/or alternative tools to accurately record program success and CRs, to detect early drug-resistance indicators, as for STHs [49]. The need for a higher number of Kato-Katzs for accurate diagnosis as the number of previous praziquantel treatments increases is not unexpected considering the small amount of stool used in each Kato-Katz and the progressively lower egg counts. Our baseline data indicated that two Kato-Katzs had a sensitivity of >90%, whereas in contrast, praziquantel-naïve individuals (Table S3) required four Kato-Katzs for accurate predictions. This apparent conflicting result may be explained by the high number of praziquantel-naïve recruits each year, sampled after several school-based MDA rounds, lowering infection intensities through reduced force-of-infections [50], supported by Figure 3, where Kato-Katz sensitivities decrease with EPG.

*S. mansoni* infection intensities were not expected to vary with Kato-Katz sampling effort. However, one-week-post annual or biannual treatment, mean intensities decreased from day one to day three, likely due to continued daily reductions in egg excretion post treatment. This, and our discordant POC-CCA and Kato-Katzs results post treatment, raise interesting questions regarding parasite antigen and egg clearance, such as residual-egg clearance (with individuals with intensities of >100 EPG at one-week, not retreated, but negative at four-weeks), praziquantel-induced fecundity compensation and/or increased egg expulsion (with higher EPGs at one-week-day-one, than at baseline, as also observed in *S. haematobium* [51]).

In contrast, at four-weeks-post-praziquantel, positive POC-CCA results in egg negative individuals may have occurred due to juveniles unaffected by treatment, newly acquired infections, and/or worms which survived treatment, but with reduced or cessated egg production (embryostasis). Drug-induced embryostasis, has been demonstrated in *Onchocerca volvulus* [52] and *Ascaris suum* [53]. Embryostasis could explain our lower sensitivities (73%) at four-weeks-post-praziquantel than those observed in a stable, low transmission Western Kenyan region (prevalence 38.8%, sensitivity = 96%) [34]. In this Kenyan region a large proportion of individuals may be truly negative with no egg or antigen excretions. Embryostasis could significantly affect drug-resistance selection, with worms repeatedly exposed to praziquantel, without dying or being detected by standard parasitological techniques. Being impossible to sample adult worms directly, studies on worm antigens, egg production and molecular studies incorporating sibship analyses informing adult breeding numbers [54], [55] post-praziquantel treatment may elucidate this.

As intensities decrease, costs of accurate diagnoses by Kato-Katzs will rise due to greater sampling requirements. Diagnoses using urine, rather than stool, remain quicker, cheaper on labor costs, more convenient, socially acceptable and may improve compliance [35]. In low endemicity areas, pooled urine samples for POC-CCAs could reduce costs further. However, key POC-CCA limitations are their inability to detect STHs, and inaccuracy measuring infection intensities and treatment resolutions. Multiple smears from one stool (*versus* multiple stools) and FLOTAC [49] may be viable diagnostic alternatives. If one sampling day can accurately detect schistosomiasis and STH infection intensities and ERRs, it may be highly cost-effective, warranting further research. As the geographical distribution of STH infections are more homogeneous than schistosomiasis, WHO recommends surveys of smaller subsets of schools for mapping and M&E [15]. We therefore recommend widespread POC-CCA use, with Kato-Katzs performed in a subsection of schools. For drug-efficacy studies we recommend at least six Kato-Katzs or one POC-CCA, with further research on clearance dynamics of eggs and antigens post treatment needed.

### Conclusions

At least four Kato-Katzs (two smears per stool from two stools) are required for M&E, in the early years of a MDA program in a highly endemic area, increasing to six Kato-Katzs (two smears per stool from three stools) by year three. One POC-CCA is a suitable alternative to current prevalence M&E protocols, but they provide no information on STHs and limited intensity data post treatment, therefore we recommend their use for *S. mansoni* M&E with Kato-Katzs performed in a subset of schools. For drug-efficacy studies, at least six Kato-Katzs (two smears per stool from three stools) are required for accurate prevalence assessment four-weeks-post-praziquantel treatment. POC-CCAs may be a promising alternative with low specificity findings potentially due to low Kato-Katzs sensitivity, however further work is required to elucidate POC-CCA's full potential for drug-efficacy studies. Further work on improved ‘gold standards’ is required to elucidate discordant POC-CCA and Kato-Katzs results. Data on multiple Kato-Katzs from a single stool post-treatment would ascertain if accuracies of multiple days of Kato-Katzs or POC-CCAs could be matched, minimizing logistical costs without overestimating M&E success and drug efficacy, whilst retaining vitally important intensity data.

## Supporting Information

Checklist S1 **STARD checklist.**
(DOC)

Table S1 ***S. mansoni*** **infection intensity categories by six Kato-Katzs and a single POC-CCA.** Proportion of World Health Organization infection intensity categories, as measured by six Kato-Katz thick smears, which are correctly identified by a single point-of-care circulating cathodic antigen test (POC-CCA) band strength, ranging from negative to three. Tests were performed pre-treatment (Baseline), one-week-post- (1 Wk), four-weeks-post- (4 Wks), six-months-post- (6 Mths) and six-months-one-week-post- (6 Mths 1 Wk) praziquantel treatment. Percentage of tests correctly identified in parentheses.
(DOCX)

Table S2 **Accuracy of one to five Kato-Katzs and a single POC-CCA for diagnosing** ***S. mansoni*** **infections over time since each child was first treated with praziquantel.** The accuracy of one to five Kato-Katz thick smears (1KK to 5KK) and a single point-of-care circulating cathodic antigen test (POC-CCA) (comparing if trace readings are counted as positive (POC-CCA-t+) or negative (POC-CCA-t−)) for detecting *S. mansoni* infection over the time since each child was first treated with praziquantel (10 time-points, in real time from the start of this study) with six Kato-Katz thick smears (6KK) as the ‘gold standard’. Sens = sensitivity, Spec = specificity, NPV = negative predictive value, PPV = positive predictive value.
(DOCX)

Table S3 **The accuracy of one to five Kato-Katzs for detecting** ***S. mansoni*** **infection over time since each child was first treated with praziquantel.** The accuracy of one to five Kato-Katz thick smears (1KK to 5KK) for detecting *S. mansoni* infection over time since each child was first treated with praziquantel (16 time-points, in real time from the start of this study) compared with the ‘gold standard’ of six Kato-Katzs (6KK), from the complete dataset of children who each had results from six Kato-Katz thick smears (n = 1065). Proportion infected given in parentheses, as measured by six Kato-Katz thick smears, although it should be noted that this is a hypothetical prevalence as the actual time-points of data collection vary from the time since each child was first exposed to praziquantel. Sens = sensitivity, Spec = specificity, NPV = negative predictive value.
(DOCX)

## References

[1] Webster JP, Molyneux D, Hotez P, Fenwick A (2014) The contribution of mass drug administration to global health – past, present and future. Philos Trans R Soc Lond B Biol Sci 369: 1471–2970. 10.1098/rstb.2013.0434 PMC4024227 24821920

[2] World Health Organization (2012). Accelarating work to overcome the global impact of neglected tropical diseases – A roadmap for implementation. Available: [http://whqlibdoc.who.int/hq/2012/WHO_HTM_NTD_2012.1_eng.pdf?ua=1], accessed: 26 March 2014.

[3] (2012) London Declaration on Neglected Tropical Diseases. http://www.who.int/neglected_diseases/London_Declaration_NTDs.pdf?ua=1.

[4] Gomes LI, Enk MJ, Rabello A (2014) Diagnosing schistosomiasis: where are we? Rev Soc Bras Med Trop 47: 3–11. 24553804 10.1590/0037-8682-0231-2013

[5] Bergquist R, Johansen MV, Utzinger J (2009) Diagnostic dilemmas in helminthology: what tools to use and when? Trends Parasitol 25: 151–156. 19269899 10.1016/j.pt.2009.01.004

[6] Brooker S, Kabatereine NB, Gyapong JO, Stothard JR, Utzinger J (2009) Rapid mapping of schistosomiasis and other neglected tropical diseases in the context of integrated control programmes in Africa. Parasitology 136: 1707–1718. 19450373 10.1017/S0031182009005940 PMC2777245

[7] Stothard JR, Chitsulo L, Kristensen TK, Utzinger J (2009) Control of schistosomiasis in sub-Saharan Africa: progress made, new opportunities and remaining challenges. Parasitology 136: 1665–1675. 19814845 10.1017/S0031182009991272

[8] Utzinger J, Raso G, Brooker S, de Savigny D, Tanner M, et al (2009) Schistosomiasis and neglected tropical diseases: towards integrated and sustainable control and a word of caution. Parasitology 136: 1859–1874. 19906318 10.1017/S0031182009991600 PMC2791839

[9] Utzinger J, N'Goran EK, Caffrey CR, Keiser J (2011) From innovation to application: social-ecological context, diagnostics, drugs and integrated control of schistosomiasis. Acta Trop 120 Suppl 1: S121–137. 20831855 10.1016/j.actatropica.2010.08.020

[10] Knopp S, Stothard JR, Rollinson D, Mohammed KA, Khamis IS, et al (2013) From morbidity control to transmission control: time to change tactics against helminths on Unguja Island, Zanzibar. Acta Trop 128: 412–422. 21586268 10.1016/j.actatropica.2011.04.010

[11] Rollinson D, Knopp S, Levitz S, Stothard JR, Tchuem Tchuente LA, et al (2013) Time to set the agenda for schistosomiasis elimination. Acta Trop 128: 423–440. 22580511 10.1016/j.actatropica.2012.04.013

[12] Albonico M, Engels D, Savioli L (2004) Monitoring drug efficacy and early detection of drug resistance in human soil-transmitted nematodes: a pressing public health agenda for helminth control. Int J Parasitol 34: 1205–1210. 15491582 10.1016/j.ijpara.2004.08.001

[13] Hotez PJ, Molyneux DH, Fenwick A, Kumaresan J, Sachs SE, et al (2007) Control of neglected tropical diseases. N Engl J Med 357: 1018–1027. 17804846 10.1056/NEJMra064142

[14] Katz N, Chaves A, Pellegrino J (1972) A simple device for quantitative stool thick-smear technique in schistosomiasis mansoni. Rev Inst Med Trop São Paulo 14: 397–400. 4675644

[15] World Health Organization (2002). Prevention and Control of Schistosomiasis and Soil-Transmitted Helminthiasis. Technical Series Report 912. Available: [http://whqlibdoc.who.int/trs/WHO_TRS_912.pdf], accessed: 15th May 2014. 12592987

[16] Engels D, Sinzinkayo E, Gryseels B (1996) Day-to-day egg count fluctuation in *Schistosoma mansoni* infection and its operational implications. Am J Trop Med Hyg 54: 319–324. 8615440 10.4269/ajtmh.1996.54.319

[17] Vennervald BJ, Ouma JH, Butterworth AE (1998) Morbidity in schistosomiasis: assessment, mechanisms and control. Parasitol Today 14: 385–390. 17040825 10.1016/s0169-4758(98)01311-8

[18] Utzinger J, Booth M, N'Goran EK, Műller I, Tanner M, et al (2001) Relative contribution of day-to-day and intra-specimen variation in faecal egg counts of *Schistosoma mansoni* before and after treatment with praziquantel. Parasitology 122: 537–544. 11393827 10.1017/s0031182001007752

[19] Booth M, Vounatsou P, N'Goran EK, Tanner M, Utzinger J (2003) The influence of sampling effort and the performance of the Kato-Katz technique in diagnosing *Schistosoma mansoni* and hookworm co-infections in rural Côte d'Ivoire. Parasitology 127: 525–531. 14700188 10.1017/s0031182003004128

[20] Knopp S, Rinaldi L, Khamis IS, Stothard JR, Rollinson D, et al (2009) A single FLOTAC is more sensitive than triplicate Kato-Katz for the diagnosis of low-intensity soil-transmitted helminth infections. Trans R Soc Trop Med Hyg 103: 347–354. 19168197 10.1016/j.trstmh.2008.11.013

[21] da Frota SM, Carneiro TR, Queiroz JA, Alencar LM, Heukelbach J, et al (2011) Combination of Kato-Katz faecal examinations and ELISA to improve accuracy of diagnosis of intestinal schistosomiasis in a low-endemic setting in Brazil. Acta Trop 120 Suppl 1: S138–141. 20522322 10.1016/j.actatropica.2010.05.007

[22] Enk MJ, Lima AC, Drummond SC, Schall VT, Coelho PM (2008) The effect of the number of stool samples on the observed prevalence and the infection intensity with *Schistosoma mansoni* among a population in an area of low transmission. Acta Trop 108: 222–228. 18973744 10.1016/j.actatropica.2008.09.016

[23] Siqueira LM, Coelho PM, Oliveira AA, Massara CL, Carneiro NF, et al (2011) Evaluation of two coproscopic techniques for the diagnosis of schistosomiasis in a low-transmission area in the state of Minas Gerais, Brazil. Mem Inst Oswaldo Cruz 106: 844–850. 22124557 10.1590/s0074-02762011000700010

[24] Lin DD, Liu JX, Liu YM, Hu F, Zhang YY, et al (2008) Routine Kato-Katz technique underestimates the prevalence of *Schistosoma japonicum*: a case study in an endemic area of the People's Republic of China. Parasitol Int 57: 281–286. 18485807 10.1016/j.parint.2008.04.005

[25] Cesari IM, Ballen DE, Mendoza L, Matos C (2005) Detection of *Schistosoma mansoni* membrane antigens by immunoblot analysis of sera of patients from low-transmission areas. Clin Diagn Lab Immunol 12: 280–286. 15699423 10.1128/CDLI.12.2.280-286.2005 PMC549304

[26] Grenfell RF, Martins W, Enk M, Almeida A, Siqueira L, et al (2013) *Schistosoma mansoni* in a low-prevalence area in Brazil: the importance of additional methods for the diagnosis of hard-to-detect individual carriers by low-cost immunological assays. Mem Inst Oswaldo Cruz 108. 10.1590/S0074-02762013000300011 PMC4005562 23778663

[27] Grenfell RF, Coelho PM, Taboada D, de Mattos AC, Davis R, et al (2014) Newly established monoclonal antibody diagnostic assays for *Schistosoma mansoni* direct detection in areas of low endemicity. PloS One 9: e87777. 24498191 10.1371/journal.pone.0087777 PMC3909226

[28] Grenfell R, Harn DA, Tundup S, Da'dara A, Siqueira L, et al (2013) New approaches with different types of circulating cathodic antigen for the diagnosis of patients with low *Schistosoma mansoni* load. PLoS Negl Trop Dis 7: e2054. 23469295 10.1371/journal.pntd.0002054 PMC3585039

[29] van Lieshout L, Panday UG, de Jonge N, Krijger FW, Oostburg BF, et al (1995) Immunodiagnosis of schistosomiasis mansoni in a low endemic area in Surinam by determination of the circulating antigens CAA and CCA. Acta Trop 59: 19–29. 7785523 10.1016/0001-706x(94)00084-e

[30] Van Dam GJ. Schistosomiasis diagnosis by circulating antigen detection: from lab-based ultimate UCP-lateral flow diagnostic to field-available point-of-care rapid test; 2012; Rio de Janeiro, Brazil.

[31] van Lieshout L, Polderman AM, Deelder AM (2000) Immunodiagnosis of schistosomiasis by determination of the circulating antigens CAA and CCA, in particular in individuals with recent or light infections. Acta Trop 77: 69–80. 10996122 10.1016/s0001-706x(00)00115-7

[32] Stothard JR, Kabatereine NB, Tukahebwa EM, Kazibwe F, Rollinson D, et al (2006) Use of circulating cathodic antigen (CCA) dipsticks for detection of intestinal and urinary schistosomiasis. Acta Trop 97: 219–228. 16386231 10.1016/j.actatropica.2005.11.004

[33] Coulibaly JT, Knopp S, N'Guessan NA, Silué KD, Fűrst T, et al (2011) Accuracy of urine circulating cathodic antigen (CCA) test for *Schistosoma mansoni* diagnosis in different settings of Côte d'Ivoire. PLoS Negl Trop Dis 5: e1384. 22132246 10.1371/journal.pntd.0001384 PMC3222626

[34] Shane HL, Verani JR, Abudho B, Montgomery SP, Blackstock AJ, et al (2011) Evaluation of urine CCA assays for detection of *Schistosoma mansoni* infection in Western Kenya. PLoS Negl Trop Dis 5: e951. 21283613 10.1371/journal.pntd.0000951 PMC3026766

[35] Colley DG, Binder S, Campbell C, King CH, Tchuem Tchuenté LA, et al (2013) A five-country evaluation of a point-of-care circulating cathodic antigen urine assay for the prevalence of *Schistosoma mansoni* . Am J Trop Med Hyg 88: 426–432. 23339198 10.4269/ajtmh.12-0639 PMC3592520

[36] Koukounari A, Donnelly CA, Moustaki I, Tukahebwa EM, Kabatereine NB, et al (2013) A latent Markov modelling approach to the evaluation of circulating cathodic antigen strips for schistosomiasis diagnosis pre- and post-praziquantel treatment in Uganda. PLoS Comput Biol 9: e1003402. 24367250 10.1371/journal.pcbi.1003402 PMC3868541

[37] Adriko M, Standley CJ, Tinkitina B, Tukahebwa EM, Fenwick A, et al (2014) Evaluation of circulating cathodic antigen (CCA) urine-cassette assay as a survey tool for *Schistosoma mansoni* in different transmission settings within Bugiri District, Uganda. Acta Trop 10.1016/j.actatropica.2014.04.001 24727052

[38] Standley CJ, Lwambo NJS, Lange CN, Kariuki HC, Adriko M, et al (2010) Performance of circulating cathodic antigen (CCA) urine-dipstick for rapid detection of intestinal schistosomiasis in schoolchildren from shoreline communities of Lake Victoria. Parasit Vectors 3: 7. 20181101 10.1186/1756-3305-3-7 PMC2828997

[39] Koukounari A, Webster JP, Donnelly CA, Bray BC, Naples J, et al (2009) Sensitivities and specificities of diagnostic tests and infection prevalence of *Schistosoma haematobium* estimated from data on adults in villages northwest of Accra, Ghana. Am J Trop Med Hyg 80: 435–441. 19270295 PMC2726788

[40] Ashton RA, Stewart BT, Petty N, Lado M, Finn T, et al (2011) Accuracy of circulating cathodic antigen tests for rapid mapping of *Schistosoma mansoni* and *S. haematobium* infections in Southern Sudan. Trop Med Int Health 16: 1099–1103. 21692957 10.1111/j.1365-3156.2011.02815.x

[41] Coulibaly JT, N'Gbesso YK, Knopp S, N'Guessan NA, Silué KD, et al (2013) Accuracy of urine circulating cathodic antigen test for the diagnosis of *Schistosoma mansoni* in preschool-aged children before and after treatment. PLoS Negl Trop Dis 7: e2109. 23556011 10.1371/journal.pntd.0002109 PMC3605147

[42] Kabatereine NB, Brooker S, Koukounari A, Kazibwe F, Tukahebwa EM, et al (2007) Impact of a national helminth control programme on infection and morbidity in Ugandan schoolchildren. Bull World Health Organ 85: 91–99. 17308729 10.2471/BLT.06.030353 PMC2174620

[43] Lamberton PHL, Hogan SC, Kabatereine NB, Fenwick A, Webster JP (2010) *In vitro* praziquantel test capable of detecting reduced *in vivo* efficacy in *Schistosoma mansoni* human infections. Am J Trop Med Hyg 83: 1340–1347. 21118946 10.4269/ajtmh.2010.10-0413 PMC2990056

[44] Landis JR, Koch GG (1977) The measurement of observer agreement for categorical data. Biometrics 33: 159–174. 843571

[45] Tchuem Tchuenté LA, Kuete Fouodo CJ, Kamwa Ngassam RI, Sumo L, Dongmo Noumedem C, et al (2012) Evaluation of circulating cathodic antigen (CCA) urine-tests for diagnosis of *Schistosoma mansoni* infection in Cameroon. PLoS Negl Trop Dis 6: e1758. 22860148 10.1371/journal.pntd.0001758 PMC3409114

[46] Erko B, Medhin G, Teklehaymanot T, Degarege A, Legesse M (2013) Evaluation of urine-circulating cathodic antigen (urine-CCA) cassette test for the detection of *Schistosoma mansoni* infection in areas of moderate prevalence in Ethiopia. Trop Med Int Health 18: 1029–1035. 23590255 10.1111/tmi.12117

[47] De Clercq D, Sacko M, Vercruysse J, vanden Bussche V, Landoure A, et al (1997) Assessment of cure by detection of circulating antigens in serum and urine, following schistosomiasis mass treatment in two villages of the Office du Niger, Mali. Acta Trop 68: 339–346. 9492918 10.1016/s0001-706x(97)00111-3

[48] Zhang Y, Koukounari A, Kabatereine N, Fleming F, Kazibwe F, et al (2007) Parasitological impact of two-year preventive chemotherapy on schistosomiasis and soil-transmitted helminthiasis in Uganda. BMC Med 5: 27. 17767713 10.1186/1741-7015-5-27 PMC2014753

[49] Knopp S, Speich B, Hattendorf J, Rinaldi L, Mohammed KA, et al (2011) Diagnostic accuracy of Kato-Katz and FLOTAC for assessing anthelmintic drug efficacy. PLoS Negl Trop Dis 5: e1036. 21532740 10.1371/journal.pntd.0001036 PMC3075226

[50] French MD, Churcher TS, Gambhir M, Fenwick A, Webster JP, et al (2010) Observed reductions in *Schistosoma mansoni* transmission from large-scale administration of praziquantel in Uganda: a mathematical modelling study. PLoS Negl Trop Dis 4: e897. 21124888 10.1371/journal.pntd.0000897 PMC2990705

[51] Stete K, Krauth SJ, Coulibaly JT, Knopp S, Hattendorf J, et al (2012) Dynamics of *Schistosoma haematobium* egg output and associated infection parameters following treatment with praziquantel in school-aged children. Parasit Vectors 5: 298. 23259435 10.1186/1756-3305-5-298 PMC3558406

[52] Plaisier AP, Alley ES, Boatin BA, Van Oortmarssen GJ, Remme H, et al (1995) Irreversible effects of ivermectin on adult parasites in onchocerciasis patients in the Onchocerciasis Control Programme in West Africa. J Infect Dis 172: 204–210. 7797912 10.1093/infdis/172.1.204

[53] Kim JS, Oh DS, Ahn KS, Shin SS (2012) Effects of kimchi extract and temperature on embryostasis of *Ascaris suum* eggs. Korean J Parasitol 50: 83–87. 22451740 10.3347/kjp.2012.50.1.83 PMC3309058

[54] Criscione CD, Poulin R, Blouin MS (2005) Molecular ecology of parasites: elucidating ecological and microevolutionary processes. Mol Ecol 14: 2247–2257. 15969711 10.1111/j.1365-294X.2005.02587.x

[55] Gower CM, Gabrielli AF, Sacko M, Dembele R, Golan R, et al (2011) Population genetics of *Schistosoma haematobium*: development of novel microsatellite markers and their application to schistosomiasis control in Mali. Parasitology 138: 978–994. 21679489 10.1017/S0031182011000722

