# Supplementary material for: Sensitivity and Specificity of Multiple Kato-Katz Thick Smears and a Circulating Cathodic Antigen Test for Schistosoma mansoni Diagnosis Pre- and Post-repeated-Praziquantel Treatment
Source: PLoS Negl Trop Dis. 2014 Sep 11;8(9):e3139. doi: 10.1371/journal.pntd.0003139 (PMC4161328; doi:10.1371/journal.pntd.0003139)
Supplement: Table S1 — S. mansoni infection intensity categories by six Kato-Katzs and a single POC-CCA. Proportion of World Health Organization infection intensity categories, as measured by six Kato-Katz thick smears, which are correctly identified by a single point-of-care circulating cathodic antigen test (POC-CCA) band strength, ranging from negative to three. Tests were performed pre-treatment (Baseline), one-week-post- (1 Wk), four-weeks-post- (4 Wks), six-months-post- (6 Mths) and six-months-one-week-post- (6 Mths 1 Wk) praziquantel treatment. Percentage of tests correctly identified in parentheses. (DOCX) [file pntd.0003139.s002.docx]

**Table S1: *S. mansoni* infection intensity categories by six Kato-Katzs and a single POC-CCA.**

| **Infection Intensity (Kato-Katz)** | **POC-CCA** | **Baseline** | **1Wk** | **4Wks** | **6Mths** | **6 Mths 1Wk** |
| --- | --- | --- | --- | --- | --- | --- |
|  | **3** | **2** | **-** | **-** | **1** | **-** |
| **Heavy**  **(≥400 EPG)** | 2 | 12 | 4 | - | - | - |
|  | 1 | 2 | 5 | - | - | 1 |
|  | trace | - | 2 | - | - | - |
|  | 0 | 1 | 1 | - | - | - |
|  | Total | 17 (11.8%) | 12 (0%) | 0 | 1 (100%) | 1 (0%) |
|  | 3 | - | - | - | 4 | - |
| **Moderate**  **(100-399 EPG)** | **2** | **13** | **-** | **-** | **3** | **1** |
|  | 1 | 12 | 3 | 1 | - | 7 |
|  | trace | 1 | 10 | - | - | - |
|  | 0 | 1 | 3 | - | - | 1 |
|  | Total | 27 (48.1%) | 16 (0%) | 1 (0%) | 7 (42.9%) | 9 (11.1%) |
|  | 3 | 1 | - | - | 5 | - |
| **Low**  **(≤100 EPG)** | 2 | 8 | - | 1 | 15 | 8 |
|  | **1** | **10** | **2** | **14** | **41** | **12** |
|  | **trace** | **5** | **8** | **3** | **-** | **5** |
|  | 0 | 4 | 13 | 7 | 6 | 10 |
|  | Total | 28 (53.6%) | 23 (43.5%) | 25 (68.0%) | 67 (61.2%) | 35 (48.6%) |
|  | 3 | - | - | - | - | - |
| **No infection** | 2 | - | - | 1 | 1 | - |
|  | 1 | - | - | 8 | 17 | 5 |
|  | trace | 1 | - | 12 | - | 2 |
|  | **0** | **3** | **5** | **29** | **1** | **13** |
|  | Total | 4 (75.0%) | 5 (100%) | 50 (58.0%) | 19 (5.3%) | 20 65.0%) |

*Proportion of World Health Organization infection intensity categories, as measured by six Kato-Katz thick smears, which are correctly identified by a single point-of-care circulating cathodic antigen test (POC-CCA) band strength, ranging from negative to three. Tests were performed pre-treatment (Baseline), one-week-post- (1Wk), four-weeks-post- (4Wks), six-months-post- (6Mths) and six-months-one-week-post- (6Mths 1Wk) praziquantel treatment. Percentage of tests correctly identified in parentheses.*
