# Supplementary material for: Sensitivity and Specificity of Multiple Kato-Katz Thick Smears and a Circulating Cathodic Antigen Test for Schistosoma mansoni Diagnosis Pre- and Post-repeated-Praziquantel Treatment
Source: PLoS Negl Trop Dis. 2014 Sep 11;8(9):e3139. doi: 10.1371/journal.pntd.0003139 (PMC4161328; doi:10.1371/journal.pntd.0003139)
Supplement: Table S2 — Accuracy of one to five Kato-Katzs and a single POC-CCA for diagnosing S. mansoni infections over time since each child was first treated with praziquantel. The accuracy of one to five Kato-Katz thick smears (1KK to 5KK) and a single point-of-care circulating cathodic antigen test (POC-CCA) (comparing if trace readings are counted as positive (POC-CCA-t+) or negative (POC-CCA-t−)) for detecting S. mansoni infection over the time since each child was first treated with praziquantel (10 time-points, in real time from the start of this study) with six Kato-Katz thick smears (6KK) as the ‘gold standard’. Sens = sensitivity, Spec = specificity, NPV = negative predictive value, PPV = positive predictive value. (DOCX) [file pntd.0003139.s003.docx]

**Table S2: Accuracy of one to five Kato-Katzs and a single POC-CCA for diagnosing *S. mansoni* infections over time since each child was first treated with praziquantel**

| **Gold Standard** | **PZQ naive (n=31)** | | | | | **1Wk (n=27)** | | | | | | | **4Wks (n=63)** | | | | | | | **6Mths (n=43)** | | | | | | | **6Mths 1Wk (n=24)** | | | | | |
| --- | --- | --- | --- | --- | --- | --- | --- | --- | --- | --- | --- | --- | --- | --- | --- | --- | --- | --- | --- | --- | --- | --- | --- | --- | --- | --- | --- | --- | --- | --- | --- | --- |
| **6KK** | **Sens** | **Spec** | **NPV** | **PPV** | | **Sens** | **Spec** | | **NPV** | | **PPV** | | **Sens** | **Spec** | | **NPV** | | **PPV** | | **Sens** | **Spec** | | **NPV** | | **PPV** | | **Sens** | **Spec** | **NPV** | | | **PPV** |
| **1KK** | 89.7% (72.6-97.8) | 100% (15.8-100) | 40.0% (5.3-85.3) |  | | 84.0% (63.9-95.5) | 100% (15.8-100) | | 33.3% (4.3-77.7) | |  | | 36.4% (17.2-59.3) | 100% (91.4-100) | | 74.5% (61.0-85.3) | |  | | 47.1% (29.8-64.9) | 100% (66.4-100) | | 33.3% (16.5-54.0) | |  | | 66.7% (41.0-86.7) | 100% ((54.1-100) | 50.0% (21.1-78.9) | | |  |
| **2KK** | 100% (88.1-100) | ' | 100% (15.8-100) |  | | 88.0% (68.8-97.5) | ' | | 40.0% (5.3-85.3) | |  | | 45.5% (24.4-67.8) | ' | | 77.4% (63.8-87.7) | |  | | 64.7% (46.5-80.3) | ' | | 42.9% (21.8-66.0) | |  | | 77.8% (52.4-93.6) | ' | 60.0% (26.2-87.8) | | |  |
| **3KK** | 100% (88.1-100) | ' | 100% (15.8-100) |  | | 96.0% (79.6-99.9) | ' | | 66.7% (9.4-99.2) | |  | | 68.2% (45.1-86.1) | ' | | 85.4% (72.2-93.9) | |  | | 73.5% (55.6-87.1) | ' | | 50.0% (26.0-74.0) | |  | | 88.9% (65.3-98.6) | ' | 75.0% (34.9-96.8) | | |  |
| **4KK** | 100% (88.1-100) | ' | 100% (15.8-100) |  | | 100% (86.3-100) | ' | | 100% (15.8-100) | |  | | 72.7% (49.8-89.2) | ' | | 87.2% (74.3-95.2) | |  | | 79.4% (62.1-91.3) | ' | | 56.3% (29.9-80.2) | |  | | 100% (81.5-100) | ' | 100% (54.1-100) | | |  |
| **5KK** | 100% (88.1-100) | ' | 100% (15.8-100) |  | | 100% (86.3-100) | ' | | 100% (15.8-100) | |  | | 95.5% (77.2-99.9) | ' | | 97.6% (87.4-99.9) | |  | | 97.1% (84.7-99.9) | ' | | 90.0% (55.5-99.7) | |  | | 100% (81.5-100) | ' | 100% (54.1-100) | | |  |
| **POC-CCA-t-** | 89.7% (72.6-97.8) | 100% (15.8-100) | 40.0% (5.3-85.3) | 100% (86.8-100) | | 40.0% (21.1-61.3) | 100% (15.8-100) | | 11.8% (1.5-36.4) | | 100% (69.2-100) | | 59.1% (36.4-79.3) | 78.0% (62.4-89.4) | | 78.0% (62.4-89.4) | | 59.1% (36.4-79.3) | | 88.2% (72.5-96.7) | 11.1% (0.3-48.2) | | 20.0% (0.5-71.6) | | 78.9% (62.7-90.4) | | 44.4% (21.5-69.2) | 66.7% ((22.3-95.7) | 28.6% (8.4-58.1) | | 80.0% (44.4-97.5) | |
| **POC-CCA-t+** | 93.1% (77.2-99.2) | 100% (15.8-100) | 50.0% (6.8-93.2) | 100% (87.2-100) | | 68.0% (46.5-85.1) | 100% (15.8-100) | | 20.0% (2.5-55.6) | | 100% (80.5-100) | | 72.7% (49.8-89.3) | 56.1% (39.7-71.5) | | 79.3% (60.3-92.0) | | 47.1% (29.8-64.9) | | 88.2% (72.5-96.7) | 11.1% (0.3-48.2) | | 20.0% (0.5-71.6) | | 78.9% (62.7-90.4) | | 66.7% (41.0-86.7) | 33.3% (4.3-77.7) | 25.0% (3.2-65.1) | | 75.0% (47.6-92.7) | |
|  | **1Yr (n=45)** | | | | | **1Yr 1Wk (n=29)** | | | | | | | **1Yr 4Wks (n=130** | | | | | | | **1Yr 6Mths (n=51)** | | | | | | | **1Yr 6Mths 1Wk (n=41)** | | | | | |
|  | **Sens** | **Spec** | **NPV** | | **PPV** | **Sens** | | **Spec** | | **NPV** | | **PPV** | **Sens** | | **Spec** | | **NPV** | | **PPV** | **Sens** | | **Spec** | | **NPV** | | **PPV** | **Sens** | **Spec** | | **NPV** | | **PPV** |
| **1KK** | 77.8% (62.9-88.8) | 100% (15.8-100) | 16.7% (2.1-48.4) | |  | 92.3% (74.9-99.1) | | 100% (29.2-100) | | 60.0% (14.7-94.7) | |  | 0.9% (0-33.6) | | 100% (66.4-100) | | 69.2% (38.6-90.9) | |  | 65.9% (49.4-79.9) | | 100% (69.2-100) | | 41.7% (22.1-63.4) | |  | 66.7% (46.0-83.5) | 100% (76.8-100) | | 60.9% (38.6-80.3) | |  |
| **2KK** | 93.33% (81.7-98.6) | ' | 40.0% (5.3-85.3) | |  | 96.2% (80.4-99.9) | | ' | | 75.0% (19.4-99.4) | |  | 75.0% (19.4-99.4) | | ' | | 90.0% (55.5-99.7) | |  | 80.5% (65.1-91.2) | | ' | | 55.6% (30.8-78.5) | |  | 81.5% (61.9-93.7) | ' | | 73.7% (48.8-90.9) | |  |
| **3KK** | 100% (91.8-100) | ' | 100% (15.8-100) | |  | 100% (86.8-100) | | ' | | 100% (29.2-100) | |  | 75.0% (19.4-99.4) | | ' | | 90.0% (55.5-99.7) | |  | 87.8% (73.8-95.9) | | ' | | 66.7% (38.4-88.2) | |  | 92.6% (75.7-99.1) | ' | | 87.5% (61.8-98.4) | |  |
| **4KK** | 100% (91.8-100) | ' | 100% (15.8-100) | |  | 100% (86.8-100) | | ' | | 100% (29.2-100) | |  | 100% (39.8-100) | | ' | | 100% (66.4-100) | |  | 92.7% (80.1-98.5) | | ' | | 76.9% (46.2-95.0) | |  | 92.6% (75.7-99.1) | ' | | 87.5% (61.8-98.4) | |  |
| **5KK** | 100% (91.8-100) | ' | 100% (15.8-100) | |  | 100% (86.8-100) | | ' | | 100% (29.2-100) | |  | 100% (39.8-100) | | ' | | 100% (66.4-100) | |  | 100% (91.4-100) | | ' | | 100% (69.2-100) | |  | 100% (87.3-100) | ' | | 100% (76.8-100) | |  |
| **POC-CCA-t-** | 79.1% (64.0-90.0) | 100% (15.8-100) | 18.2% (2.3-51.8) | | 100% (89.7-100) | 15.4% (4.4-34.9) | | 100% (29.2-100) | | 12.0% (2.5-31.2) | | 100% (39.8-100) | 75.0% (19.4-99.4) | | 100% (66.4-100) | | 90.0% (55.5-99.7) | | 100% (29.2-100) | 95.1% (83.5-99.4) | | 0% (0-30.8) | | 0% (0-84.2) | | 79.6% (65.7-89.8) | 77.8% (57.7-91.4) | 78.6% (49.2-95.3) | | 64.7% (38.3-85.8) | | 87.5% (67.6-97.3) |
| **POC-CCA-t+** | 90.7% (77.9-97.4) | 50.0% (1.3-100) | 20.0% (0.5-71.6) | | 97.5% (86.8-99.9) | 65.4% (44.3-82.8) | | 100% (29.2-100) | | 25.0% (5.5-57.2) | | 100% (80.5-100) | 75.0% (19.4-99.4) | | 66.7% (30.0-92.5) | | 85.7% (42.1-99.6) | | 50.0% (11.8-88.2) | 95.1% (83.5-99.4) | | 0% (0-30.8) | | 0% (0-84.2) | | 79.6% (65.7-89.8) | 81.5% (61.9-93.7) | 78.6% (49.2-95.3) | | 68.8% (41.3-89.0) | | 88.0% (68.8-97.5) |

*The accuracy of one to five Kato-Katz thick smears (1KK to 5KK) and a single point-of-care circulating cathodic antigen test (POC-CCA) (comparing if trace readings are counted as positive (POC-CCA-t+) or negative (POC-CCA-t-)) for detecting S. mansoni infection over the time since each child was first treated with praziquantel (10 time-points, in real time from the start of this study) with six Kato-Katz thick smears (6KK) as the ‘gold standard’. Sens = sensitivity, Spec = specificity, NPV = negative predictive value, PPV = positive predictive value.*
