# Supplementary material for: Sensitivity and Specificity of Multiple Kato-Katz Thick Smears and a Circulating Cathodic Antigen Test for Schistosoma mansoni Diagnosis Pre- and Post-repeated-Praziquantel Treatment
Source: PLoS Negl Trop Dis. 2014 Sep 11;8(9):e3139. doi: 10.1371/journal.pntd.0003139 (PMC4161328; doi:10.1371/journal.pntd.0003139)
Supplement: Table S3 — The accuracy of one to five Kato-Katzs for detecting S. mansoni infection over time since each child was first treated with praziquantel. The accuracy of one to five Kato-Katz thick smears (1KK to 5KK) for detecting S. mansoni infection over time since each child was first treated with praziquantel (16 time-points, in real time from the start of this study) compared with the ‘gold standard’ of six Kato-Katzs (6KK), from the complete dataset of children who each had results from six Kato-Katz thick smears (n = 1065). Proportion infected given in parentheses, as measured by six Kato-Katz thick smears, although it should be noted that this is a hypothetical prevalence as the actual time-points of data collection vary from the time since each child was first exposed to praziquantel. Sens = sensitivity, Spec = specificity, NPV = negative predictive value. (DOCX) [file pntd.0003139.s004.docx]

**Table S3: The accuracy of one to five Kato-Katzs for detecting *S. mansoni* infection over time since each child was first treated with praziquantel.**

| **Gold Standard 6KK** | **Baseline True PZQ naïve (80.2% )** | | | **1Wk (75.8%)** | | | **4Wks (28.1%)** | | | **6Mths (79.1%)** | | | **6Mths 1Wk (71.0%)** | | |
| --- | --- | --- | --- | --- | --- | --- | --- | --- | --- | --- | --- | --- | --- | --- | --- |
|  | **Sens** | **Spec** | **NPV** | **Sens** | **Spec** | **NPV** | **Sens** | **Spec** | **NPV** | **Sens** | **Spec** | **NPV** | **Sens** | **Spec** | **NPV** |
| **1KK** | 80.2% (69.9-88.3) | 100% (83.2-100) | 55.6% (38.1-72.0) | 70.7% (59.0-80.6) | 100% (85.8-100) | 52.2% (36.9-67.1) | 42.9% (26.3-60.6) | 100% (95.9-100) | 81.7% (73.1-88.4) | 47.1% (29.8-64.9) | 100% (66.4-100) | 33.3% (16.5-54.0) | 63.6% (40.7-82.8) | 100% (66.4-100) | 52.9% (27.8-77.0) |
| **2KK** | 86.4% (77.0-93.0) | “ | 64.5% (45.4-80.8) | 74.7% (63.3-84.0) | “ | 55.8% (39.9-70.9) | 54.3% (36.6-71.2) | “ | 84.8% (76.4-91.0) | 64.7% (46.5-80.3) | “ | 42.9% (21.8-66.0) | 81.8% (59.7-94.8) | “ | 69.2% (38.6-90.9) |
| **3KK** | 92.6% (84.6-97.2) | “ | 76.9% (56.4-91.0) | 92.0% (83.4-97.0) | “ | 80.0% (61.4-92.3) | 77.1% (59.9-89.6) | “ | 91.8% (84.4-96.4) | 73.5% (55.6-87.1) | “ | 50.0% (26.0-74.0) | 90.9% (70.8-98.9) | “ | 81.8% (48.2-97.7) |
| **4KK** | 96.3% (89.6-99.2) | “ | 87.0% (66.4-97.2) | 96.0% (88.8-99.2) | “ | 88.9% (70.8-97.6) | 80.0% (63.1-91.6) | “ | 92.7% (85.6-97.0) | 79.4% (62.1-91.3) | “ | 56.3% (29.9-80.2) | 100% (84.6-100) | “ | 100% (66.4-100) |
| **5KK** | 98.8% (93.3-99.9) | “ | 95.2% (76.2-99.9) | 98.7% (92.8-99.9) | “ | 96.0% (79.6-99.9) | 97.1% (85.1-99.9) | “ | 98.9% (94.0-99.9) | 97.1% (84.7-99.9) | “ | 90.0% (55.5-99.7) | 100% (84.6-100) | “ | 100% (66.4-100) |
| **6KK** | **1Yr (74.8%)** | | | **1Yr 1Wk (70.0%)** | | | **1Yr 4Wks (9.2%)** | | | **1Yr 6Mths (80.0%)** | | | **1Yr 6Mths 1Wk (61.5%)** | | |
|  | **Sens** | **Spec** | **NPV** | **Sens** | **Spec** | **NPV** | **Sens** | **Spec** | **NPV** | **Sens** | **Spec** | **NPV** | **Sens** | **Spec** | **NPV** |
| **1KK** | 71.4% (61.4-80.1) | 100% (89.4-100) | 54.1% (40.8-66.9) | 77.9% (67.0-86.6) | 100% (89.4-100) | 66.0% (51.2-78.8) | 14.3% (0.4-57.9) | 100% (94.8-100) | 92.0% (83.4-97.0) | 62.8% (46.7-77.0) | 100% (71.5-100) | 40.7% (22.4-61.2) | 62.5% (43.7-78.9) | 100% (83.2-100) | 62.5% (43.7-78.9) |
| **2KK** | 86.7% (78.4-92.7) | “ | 71.7% (56.5-84.0) | 81.8% (71.4-89.7) | “ | 70.2% (55.1-82.7) | 71.4% (29.0-96.3) | “ | 97.2% (90.2-99.7) | 81.4% (66.6-91.6) | “ | 57.9% (33.5-79.7) | 75.0% (56.6-88.5) | “ | 71.4% (51.3-86.8) |
| **3KK** | 93.9% (87.1-97.7) | “ | 84.6% (69.5-94.1) | 94.8% (87.2-98.6) | “ | 89.2% (74.6-97.0) | 85.7% (42.1-99.6) | “ | 98.6% (92.3-99.9) | 88.4% (74.9-96.1) | “ | 68.8% (41.3-89.0) | 90.6% (75.0-98.0) | “ | 87.0% (66.4-97.2) |
| **4KK** | 95.9% (89.9-98.9) | “ | 89.2% (74.6-97.0) | 96.1% (89.0-99.2) | “ | 91.7% (77.5-98.2) | 100% (59.0-100) | “ | 100% (94.8-100) | 93.0% (80.9-98.5) | “ | 78.6% (49.2-95.3) | 93.8% (79.2-99.2) | “ | 90.9% (70.8-98.9) |
| **5KK** | 99.0% (94.4-99.9) | “ | 97.1% (84.7-99.9) | 98.7% (93.0-99.9) | “ | 97.1% (84.7-99.9) | 100% (59.0-100) | “ | 100% (94.8-100) | 100% (91.8-100) | “ | 100% (71.5-100) | 100% (89.1-100) | “ | 100% (83.2-100) |
| **6KK** | **2Yrs (51.9%)** | | | **2Yrs 1Wk (38.3%)** | | | **2Yrs 4Wks (20.4%)** | | |  | | | | | |
|  | **Sens** | **Spec** | **NPV** | **Sens** | **Spec** | **NPV** | **Sens** | **Spec** | **NPV** |  |  |  |  |  |  |
| **1KK** | 50.0% (30.6-69.4) | 100% (86.8-100) | 65.0% (48.3-79.4) | 30.4% (13.2-52.9) | 100% (90.5-100) | 69.8% (55.7-81.7) | 36.4% (10.9-69.2) | 100% (91.8-100) | 86.0% (73.3-94.2) |  |  |  |  |  |  |
| **2KK** | 64.3% (44.1-81.4) | “ | 72.2% (54.8-85.8) | 60.9% (38.5-80.3) | “ | 80.4% (66.1-90.6) | 45.5% (16.7-76.6) | “ | 87.8% (75.2-95.4) |  |  |  |  |  |  |
| **3KK** | 75.0% (55.1-89.3) | “ | 78.8% (61.1-91.0) | 78.3% (56.3-92.5) | “ | 88.1% (74.4-96.0) | 63.6% (30.8-89.1) | “ | 91.5% (79.6-97.6) |  |  |  |  |  |  |
| **4KK** | 100% (87.7-100) | “ | 100% (86.8-100) | 82.6% (61.2-95.0) | “ | 90.2% (76.9-97.3) | 63.6% (30.8-89.1) | “ | 91.5% (79.6-97.6) |  |  |  |  |  |  |
| **5KK** | 100% (87.7-100) | “ | 100% (86.8-100) | 87.0% (66.4-97.2) | “ | 92.5% (79.6-98.4) | 90.9% (58.7-99.8) | “ | 97.7% (88.0-99.9) |  |  |  |  |  |  |
| **6KK** | **3Yrs (84.4%)** | | | **3Yrs 1Wk (75.0%)** | | | **3Yrs 4Wks (20.8%)** | | |  | | | | | |
|  | **Sens** | **Spec** | **NPV** | **Sens** | **Spec** | **NPV** | **Sens** | **Spec** | **NPV** |  |  |  |  |  |  |
| **1KK** | 55.6% (35.3-74.5) | 100% (47.8-100) | 29.4% (10.3-56.0) | 73.3% (44.9-92.2) | 100% (47.8-100) | 55.6% (21.2-86.3) | 60.0% (14.7-94.7) | 100% (82.4-100) | 90.5% (69.6-98.8) |  |  |  |  |  |  |
| **2KK** | 74.1% (53.7-88.9) | “ | 41.7% (15.2-72.3) | 86.7% (59.5-98.3) | “ | 71.4% (29.0-96.3) | 60.0% (14.7-94.7) | “ | 90.5% (69.6-98.8) |  |  |  |  |  |  |
| **3KK** | 88.9% (70.8-97.6) | “ | 62.5% (24.5-91.5) | 100% (78.2-100) | “ | 100% (47.8-100) | 60.0% (14.7-94.7) | “ | 90.5% (69.6-98.8) |  |  |  |  |  |  |
| **4KK** | 88.9% (70.8-97.6) | “ | 62.5% (24.5-91.5) | 100% (78.2-100) | “ | 100% (47.8-100) | 60.0% (14.7-94.7) | “ | 90.5% (69.6-98.8) |  |  |  |  |  |  |
| **5KK** | 92.6% (75.7-99.1) | “ | 71.4% (29.0-96.3) | 100% (78.2-100) | “ | 100% (47.8-100) | 100% (47.8-100) | “ | 100% (82.4-100) |  |  |  |  |  |  |

*The accuracy of one to five Kato-Katz thick smears (1KK to 5KK) for detecting S. mansoni infection over time since each child was first treated with praziquantel (16 time-points, in real time from the start of this study) compared with the ‘gold standard’ of six Kato-Katzs (6KK), from the complete dataset of children who each had results from six Kato-Katz thick smears (n=1065). Proportion infected given in parentheses, as measured by six Kato-Katz thick smears, although it should be noted that this is a hypothetical prevalence as the actual time-points of data collection vary from the time since each child was first exposed to praziquantel. Sens = sensitivity, Spec = specificity, NPV = negative predictive value.*
